# α-Mangostin prevents diabetic cardiomyopathy by inhibiting oxidative damage and lipotoxicity through the AKT–FOXO1–CD36 pathway

**DOI:** 10.3389/fphar.2025.1566311

**Published:** 2025-04-17

**Authors:** Xue Bai, Ziqian Zhang, Miao Zhang, Jiaojiao Xu, Keting Dong, Qian Du, Lei Chen, Ping Ma, Jianhong Yang

**Affiliations:** Medical School, University of Chinese Academy of Sciences, Beijing, China

**Keywords:** diabetic cardiomyopathy, alpha-mangostin, oxidative damage, lipotoxicity, forkhead box class O1

## Abstract

**Introduction:**

Diabetic cardiomyopathy (DCM), a cardiac complication of diabetes, is the main cause of the high prevalence of heart failure and associated mortality in diabetic patients. Oxidative stress and lipid metabolism disorder-induced myocardial cell damage are part of the pathogenesis of DCM. In this study, we investigated the effects of alpha-mangostin (A-MG), a natural antioxidant extracted from mangosteen peel, on *in vitro* and *in vivo* DCM models.

**Methods:**

H9C2 rat cardiomyocytes were treated with high glucose (HG) and palmitic acid (PA) for 24 h to establish an *in vitro* DCM cell model. Cell viability and cytotoxicity were evaluated after treatment with varying concentrations of A-MG (0.3, 1, 3, 9, or 27 μM) using Cell Counting Kit-8 (CCK8) and lactate dehydrogenase (LDH) assays. Flow cytometry assessment was used to detect apoptosis. Molecular mechanisms were investigated through transcriptome analysis, quantitative PCR (RT-qPCR), and Western blotting. Type 2 diabetic (T2D) mice, induced by feeding a high-fat diet (HFD) combined with low-dose streptozotocin (STZ), received either vehicle, low-dose A-MG (100 mg/kg/d), or high-dose A-MG (200 mg/kg/d) for 6 weeks. Cardiac function was assessed by echocardiography. H&E and Masson’s staining were used to evaluate cardiac tissue structure and fibrosis, and Western blotting was used to evaluate myocardial protein expression.

**Results:**

In HG/F-induced H9C2 cells, A-MG (1 and 3 μM) significantly increased cell viability (p < 0.01) and reduced LDH release (p < 0.05). A-MG (3 μM) attenuated lipid accumulation (p < 0.05), normalized mitochondrial membrane potential (p < 0.01), and inhibited reactive oxygen species (ROS) generation (p < 0.05), malondialdehyde (MDA) production (p < 0.01), and apoptosis (p < 0.05). A-MG also inhibited the nuclear translocation of Forkhead box class O1 (FOXO1) (p < 0.05); reduced the expression of CD36 (p < 0.05), PPARα (p < 0.01), and CPT1β (p < 0.05) proteins; enhanced superoxide dismutase (SOD) activity (p < 0.05); and upregulated nuclear factor erythroid 2-related factor 2 (Nrf2) (p < 0.01), HO-1 (p < 0.05), and SOD2 (p < 0.05) protein expression levels. Further investigation in HG/F-induced H9C2 cells indicated that A-MG inhibits the uptake of fatty acids (FAs) by regulating the AKT/FOXO1/CD36 signaling pathway, reduces excessive β-oxidation of FAs mediated by PPARα/CPT1β through the inhibition of FOXO1 nuclear translocation, and stimulates the AKT/Nrf2/HO-1 signaling pathway to increase the cellular antioxidant capacity. In diabetic mice, low-dose A-MG treatment increased anti-oxidative stress capacity, decreased myocardial lipid accumulation, reduced fibrosis and cardiomyocyte apoptosis, and improved left ventricular contractile function.

**Conclusion:**

Using both *in vitro* and *in vivo* DCM models, our study demonstrates that A-MG reduces lipid accumulation and excessive mitochondrial β-oxidation while enhancing antioxidant capacity. These results suggest that A-MG may be a novel therapeutic option for DCM.

## 1 Introduction

Diabetes mellitus (DM) is a highly prevalent chronic metabolic disease associated with high morbidity and mortality. A 2019 report by the International Diabetes Federation indicated a global prevalence of diabetes of 9.3%, which is expected to increase to 10.9% by 2045 (Saeedi et al., 2019). Diabetic cardiomyopathy (DCM) is defined as a unique abnormality of ventricular structure and function in diabetic patients independent of high blood pressure, coronary heart disease, or other potential cardiogenic etiology (Paolillo et al., 2019). The main pathological mechanisms of DCM include oxidative stress, cardiometabolic disorders, myocardial hypertrophy and fibrosis, and ventricular diastolic or systolic dysfunction (Parim et al., 2019; Jia et al., 2018). Although there has been a substantial increase in clinical research focused on the prevention and treatment of DCM during the past 10 years, the fundamental mechanisms that drive its development remain elusive. Moreover, there is a shortage of specific clinical treatment approaches for this condition (Mittal et al., 2021; Eo et al., 2017).

Under physiological conditions, the oxidation of fatty acids (FAs) serves as the primary energy source for the adult heart, providing approximately 70% of ATP, with the remaining energy supply coming from glucose, ketone bodies, and branched-chain amino acids (Wang et al., 2021). However, in obese and diabetic individuals, insulin resistance and hyperlipidemia result in a significantly reduced ability to use glucose for energy production, and cardiomyocytes become almost entirely dependent on FAs for energy supply. Paralleling this metabolic change, the disequilibrium between the uptake and metabolism of FAs promotes cardiac accumulation of lipids, including FA-derived ceramides and diacylglycerols, leading to lipotoxic cardiomyopathy. The ensuing alteration in the metabolism of cardiac cells is compounded by mitochondrial dysfunction, leading to excess production of reactive oxygen species (ROS) and oxidative damage of DNA, proteins, and lipids. This ultimately causes cardiac structural and functional deficits, eventually resulting in heart failure (Ritchie and Abel, 2020; Mahajan et al., 2015; Carpentier, 2018; Pham et al., 2023). Therefore, balancing lipid metabolism to mitigate oxidative stress holds great promise as a strategy to enhance cardiac efficiency in patients with DCM.

The CD36 protein plays a significant role in the uptake of FAs by myocardial cells. CD36, also referred to as fatty acid translocase, belongs to the class B scavenger receptor family (Luiken et al., 2016). Approximately 70% of the FAs that provide energy to myocardial tissue are internalized via CD36 (Kim and Dyck, 2016). Studies have shown that under insulin stimulation, CD36 translocates from the endosomal compartment to plasma membrane lipid rafts, a process regulated through the phosphoinositide 3-kinase (PI3K)-AKT and adenosine monophosphate kinase (AMPK) signaling pathways (Samovski et al., 2018).

Forkhead box class O (FOXO) transcription factors belong to the FOX superfamily, a group of evolutionarily conserved transcriptional regulators comprising 19 members (Chistiakov et al., 2017). The transcriptional regulation of FOXO1 is of vital importance as it is involved in the regulation of apoptosis, proliferation, metabolism, and other biological processes (Shukla et al., 2016). In the heart, AKT-dependent phosphorylation and protein phosphatase 2A (PP2A)-dependent dephosphorylation of FOXO1 regulate its nuclear localization, which impacts the expression of target genes related to apoptosis, lipid metabolism, and oxidative stress (Gross et al., 2008; Sengupta et al., 2011). Studies have shown that in the setting of insulin resistance, the activation of the AKT/FOXO1 signaling pathway is reduced; consequently, FOXO1 activity is enhanced, which promotes hepatic gluconeogenic gene expression, leading to increased blood glucose levels (Accili and Arden, 2004). It has been reported that chronic insulin stimulation induces CD36 mRNA/protein expression by activating the transcriptional activity of FOXO1 (Chistiakov et al., 2017).

α-Mangostin (A-MG) is a type of xanthone compound extracted from the mangosteen (Garcinia mangostana) fruit peel. A-MG has potent antioxidant activity, derived from the three phenolic hydroxyl groups attached to its conjugated ring structure, which provide electrons or hydrogen atoms to neutralize or stabilize free radicals (Alam et al., 2023; Yahyazadeh et al., 2024). In recent years, it has been demonstrated that natural products with antioxidant properties can alleviate DCM in animal models and cardiomyocytes *in vitro*. For instance, sulforaphane, curcumin, and fucoxanthin inhibit myocardial oxidative damage in type 2 diabetic (T2D) mice by activating nuclear factor erythroid 2-related factor 2 (Nrf2), a master regulator of antioxidant gene expression, resulting in reduced myocardial fibrosis, improved cardiac function, and improved DCM symptomatology (Bai et al., 2013; Wu et al., 2022b; Zheng et al., 2022). Studies confirmed that A-MG has broad therapeutic potential, as exemplified by anti-obesity (Choi et al., 2015), anti-cancer (Borzdzilowska and Bednarek, 2022), anti-inflammatory (Chen et al., 2008), hepatoprotective (Rodniem et al., 2019), and cardioprotective (Eisvand et al., 2022) effects. A study showed that in rats fed a high-fat diet (HFD), supplementation with the rind of G. mangostana fruit significantly reduced left ventricular inflammatory cell infiltration and perivascular collagen deposition (John et al., 2021). Soetikno et al. (2020) demonstrated that A-MG administration reduced cardiac hypertrophy and fibrosis and improved biochemical parameters related to liver and kidney function in diabetic rats, with beneficial effects comparable to those of metformin. Consistent with the abovementioned evidence, Lee et al. (2018) reported that the addition of 5 μM A-MG restored insulin signaling and reduced streptozotocin (STZ)-induced pancreatic β-cell damage by activating the PI3K/AKT signaling pathway.

The objective of this study was to determine the mechanism of action of A-MG in both *in vitro* and *in vivo* DCM models, providing a foundational background for its therapeutic application in the prevention and treatment of DCM.

## 2 Materials and methods

### 2.1 Cell culture

H9C2 embryonic rat heart-derived cell lines, obtained from Procell (CL-0089, Wuhan, China), were grown and maintained in Dulbecco’s modified Eagle’s medium (DMEM, Gibco, United States) containing 10% fetal bovine serum (FBS, C04001-500, VivaCell, China) at 37°C with 5% CO_2_.

In the first series of *in vitro* experiments, H9C2 cells were treated with 30 mmol/L glucose (Macklin, China) and different concentrations of palmitic acid (PA, Sigma, United States; 50, 150, 250, 350, or 500 μM) for 12, 24, 36, or 48 h. Different concentrations of A-MG (0.3, 1, 3, 9, or 27 μM) were then added to cells maintained under modeling conditions of 30 mmol/L glucose and 250 μM PA (HG/F) for 24 h. In further experiments, a concentration of 3 μmol/L A-MG was selected. In the second series of *in vitro* experiments, H9C2 cells were treated with 5.5 mM glucose (NC group), 30 mM glucose with 250 μM PA (HG/F group), or 30 mM glucose with 250 μM PA plus 3 μM A-MG (A-MG group) 24 h before harvest. In the third part of the experimental series, LY294002 (MEM, HY-10108, China), an AKT inhibitor, was added at a concentration of 10 μM to H9C2 cells divided into NC, HG/F, HG/F plus LY294002, A-MG, and A-MG plus LY294002 groups. Final experiments were conducted in cells modified with a stable FOXO1 overexpression system (FOXO1-OE; Gene ID: 84482, OBiO Technology, Shanghai, China) grouped into NC, HG/F, FOXO1-OE plus HG/F, A-MG, and FOXO1-OE plus A-MG groups.

### 2.2 Animals and treatment

Eight-week-old male C57BL/6J wild-type mice (n = 20, 21 ± 1 g) were obtained from Beijing HFK Bioscience Co., Ltd. (Beijing, China). The mice were assigned to a control group (n = 5) or a DCM model group (n = 15); the latter received an HFD (60% fat, 20% protein, and 20% carbohydrates; XIAOSHUYOUTAI, Beijing, China) 4 weeks, followed by an intraperitoneal injection of 45 mg/kg streptozotocin (STZ, S1030, Sigma-Aldrich, United States; formulated in 0.1 M citrate buffer; pH = 4.5) for five consecutive days to induce type 2 diabetes (Yuan et al., 2022). Two weeks after concluding the STZ injection schedule, the successful establishment of a T2D mouse model was defined as a 6-h fasting blood glucose (FBG) > 11.1 mmol/L. In the control group, the mice received intraperitoneal injections of the STZ vehicle (0.1 M of citrate buffer; pH 4.5) daily for 5 days. Diabetic mice were then randomly divided into three groups. The model group was gavaged with the vehicle solution (0.05% hydroxyethylcellulose; C8621, Solarbio, Beijing, China), while A-MG dissolved in 0.05% hydroxyethylcellulose was administered at 100 mg/kg/d (low-dose group) or 200 mg/kg/d (high-dose group) for 6 weeks. Purified A-MG (≥98%) was obtained from Wuhan ChemFaces Biochemical Co., Ltd. (Wuhan, China). Body weight and FBG (6-h fasting) were measured once a week, while food and water intake were measured thrice a week. After echocardiography, the mice were anesthetized with phenobarbital and euthanized by spinal cord dislocation.

Throughout the entire experimental duration, the mice were housed in an environment maintained at 24°C ± 1°C, with a 12 h/12 h light/dark cycle and unrestricted access to both food and water. The animal experimental protocols in this study were approved by the Animal Care and Use Committee of the University of Chinese Academy of Sciences (approval no. UCAS-A-20220923).

### 2.3 Cell proliferation and cytotoxicity assays

A Cell Counting Kit-8 assay (CCK8; CK001, Lablead Biotech, Beijing, China) was used to measure cell proliferation rates. A lactate dehydrogenase assay (LDH; BC0658, Solarbio, China) was used to evaluate cytotoxicity. Both assays were conducted following the instructions provided by the reagents’ manufacturers, and untreated cells cultured in a normal medium were used as the control group.

### 2.4 Detection of intracellular ROS

H9C2 cells were incubated with 10 μM of 2′, 7′-dichlorofluorescin diacetate (DCFH-DA; CA1410, Solarbio, China) diluted in serum-free DMEM at 37°C for 30 min. After two washes with PBS, cell fluorescence was assessed using a FACSCanto II flow cytometer (BD Biosciences, NJ, United States).

### 2.5 Mitochondrial membrane potential detection

5,5′,6,6′-Tetrachloro-1,1′,3,3′-tetraethyl-imidacarbocyanine (JC-1) staining was used to detect changes in mitochondrial membrane potential, as previously described (Xu et al., 2024a). H9C2 cells were incubated with the JC-1 reagent (J22202, Lablead Biotech, China), according to the reagent manufacturer’s instructions, and viewed under an inverted fluorescence microscope.

### 2.6 Apoptosis assay

H9C2 cells were washed with PBS, harvested with trypsin solution without EDTA, and centrifuged. Following three washes in pre-cooled PBS, the cells were resuspended in 500 μL of 1-x buffer containing 5 μL of Annexin V/FITC (CA1020, Solarbio, China) and incubated at 37°C in the dark for 5 min. A 5-μL propidium iodide (PI) solution was then added for another 5 min. The cells were then filtered through a 70-μm cell strainer and immediately analyzed by flow cytometry. FlowJo software was used to analyze the apoptosis rate.

### 2.7 Detection of SOD activity

H9C2 cells were washed twice with PBS and collected with superoxide dismutase (SOD) extraction reagents (BC0175, Solarbio, China), and the levels of SOD in H9C2 cells were estimated according to the manufacturer’s instructions.

### 2.8 Malondialdehyde assay

A colorimetric lipid peroxidation assay kit (Beyotime, S0131S, China), based on the reaction of malondialdehyde (MDA) with thiobarbituric acid to produce a red chromogen, was used according to the manufacturer’s instructions to measure MDA levels, indicative of lipid peroxidation, in H9C2 cell lysates and mouse serum.

### 2.9 RNA sequencing and analysis

RNA was extracted from H9C2 cells using the TRIzol reagent (BS259A, Biosharp, Beijing, China). The BGISEQ platform (BGI-Shenzhen, China) was used to perform RNA sequencing and sequence quality control of cells in the NC, HG/F, and A-MG groups. BGI’s Dr. Tom multi-omics data mining system was used to perform gene set enrichment analysis (GSEA) and generate heatmaps of transcriptomics data. The Kyoto Encyclopedia of Genes and Genomes (KEGG) database was queried for gene annotation using RNA sequencing data.

### 2.10 Quantitative real-time PCR

Total cellular RNA was extracted using TRIzol (BS259A, Biosharp, China), following the manufacturer’s instructions. The quality of the extracted RNA was assessed using a NanoDrop 2000 Analyzer (Thermo Fisher Scientific, DE, United States). Subsequently, 1 μg of total RNA was reverse-transcribed into cDNA using a Hifair III 1st Strand cDNA Synthesis SuperMix for qPCR kit (11141ES60, Yeasen Biotechnology, Beijing, China). PCR amplification was carried out using TransStart Top Green qPCR SuperMix (+Dye II) (AQ132-24, TransGen Biotech, Beijing, China), as previously described (Zhang et al., 2024). Quantification was conducted using the 2^(-△△Ct)^ method, with β-actin used as a control. The primer sequences are presented in Table 1.

**TABLE 1:** Primers for RT-qPCR.

Gene symbol	NCBI reference Sequence	Forward primer (5′→3′)	Reverse primer (5′→3′)
Nrf2	NM_031789.3	TGC​CTT​CCT​CTG​CTG​CCA​TTA​GT	ACC​GTG​CCT​TCA​GTG​TGC​TTC​T
HO-1	NM_012580.2	TGC​TCG​CAT​GAA​CAC​TCT​GGA​GAT	ATG​GCA​TAA​ATT​CCC​ACT​GCC​ACG
SOD2	NM_017051.2	TAG​GCT​AAG​GAT​GGA​TGG​A	CAGAGGCACAATGTCACT
CD36	NM_031561.2	ATA​ACT​GGA​TTC​ACT​CTA​CAG​TTT​GC	GAT​CTG​CAA​GCA​CAG​TAT​GAA​ATC
PPARα	NM_013196.2	TCG​TGG​AGT​CCT​GGA​ACT​GA	GAG​TTA​CGC​CCA​AAT​GCA​CC
CPT1β	NM_013200.2	GAG​ACT​GTG​CGT​TCC​TGT​ACT​AGC	TTG​GAG​ACG​ATG​TAG​AGG​CAG​AAG​A

### 2.11 Western blotting

Total protein was extracted from heart tissue and H9C2 cells using a RIPA lysis buffer system (R0010, Solarbio, China) containing 1 mM of the protease inhibitor (P0100, Solarbio) and 1 mM of the phosphatase inhibitor (P1260, Solarbio, China) on ice for 20 min. A BCA protein assay kit (B5001, Lablead Biotech, China) was used to measure protein concentrations. Equal amounts of protein (30–40 μg) were separated using 10%–12% SDS-PAGE and transferred onto PVDF membranes. The membranes were blocked with 5% skim milk and prepared with TBST at room temperature for 2 h. Primary antibodies were incubated at 4°C overnight. These included BCL-2 (68103-1), ACTB (81115-1-RR), Nrf2 (16396-1-AP), CPT1β (22170-1), ACADM (55210-1), and PPARα (66826-1), all purchased from Proteintech (Wuhan, China); BAX (#2772), SOD2 (#13141), p-FOXO1 (#9461), FOXO1 (#2880), and caspase9 (#9508), purchased from Cell Signaling Technology (Inc., MA, United States); cleaved-caspase 3 (WL01992), cleaved-caspase 9 (WL01838), and HO-1 (WL02400), purchased from Wanleibio (Shenyang, China); CD36 (bs-8873R) and p-AKT (bs-0876R), purchased from Bioss (Beijing, China); and AKT (179463, Abcam). Secondary antibody (goat anti-rabbit IgG(H + L)-HRP; S0101, Lablead Biotech, China), diluted 1:5000, was incubated at room temperature for 1 h. An enhanced chemiluminescence kit (#170–5060, Bio-Rad, Hercules, CA, United States) was used to detect protein signals. ImageJ software (NIH, Bethesda, MA, United States) was used for densitometric analysis of protein bands. β-actin (ACTB) was used as a reference to calculate the relative expression of target proteins.

### 2.12 Cell staining

One-step Hoechst staining (40744ES60, Yeasen Biotechnology, China) was performed on H9C2 cells to detect apoptosis. For lipid droplet staining, H9C2 cells were fixed with 4% paraformaldehyde and subsequently treated with 0.5% Triton X-100. A BODIPY 493/503 solution (4 μM of final concentration) was added to cells at room temperature for 10 min. The cells were then washed with distilled water and imaged under a fluorescence microscope (Leica Microsystems, Wetzlar, Germany).

### 2.13 Immunofluorescence

Cells were inoculated in confocal dishes, fixed in 4% paraformaldehyde for 15 min, washed three times with PBS, then incubated with 0.25% Triton X-100 for 10 min, and again washed three times with PBS. Goat serum (10%) was enclosed at room temperature for 60 min. The FOXO1 antibody (1:300) was dissolved in 0.1% BSA solution and incubated at 4°C overnight. YSFluor™ 488 Goat Anti-Rabbit lgG (H + L) antibody (33106ES60, Yeasen Biotechnology, China) (1:300) was dissolved in a 0.1% BSA solution, incubated at room temperature for 60 min, and washed three times with PBS. It was treated with an antifade mounting medium containing DAPI (ZLI-9557, ZSGB-BIO, Beijing, China) before observation. Images were captured using a laser scanning confocal microscope (Carl Zeiss LSM 880, Jena, Germany).

### 2.14 Lentiviral vector construction and subsequent infection

A lentiviral vector (OBiO, Shanghai, China) was used to construct a FOXO1 overexpression cell system, with a working concentration of 1 mg/mL of poly-plus (OBiO) employed to transduce H9C2 cells. After 72 h, GFP fluorescence was verified using fluorescence microscopy, and transduction efficiency was verified using RT-qPCR or Western blotting.

### 2.15 Echocardiography

Transthoracic echocardiography was performed in mice under 0.8% isoflurane anesthesia using a Vevo 2100 Imaging System (FUJIFILM VisualSonics, Inc., Canada) at the animal center of Capital Medical University (Beijing, China). M-mode images were obtained to measure left ventricular internal end-diastolic diameter (LVIDd), left ventricular ejection fraction (LVEF), and fractional shortening (FS). Surface electrocardiogram and heart rates were additionally recorded.

### 2.16 Serum BNP, CK-MB, and cTnⅠ measurements

After the intervention, blood samples were collected from the orbital sinus of the mice before euthanasia. The samples were kept overnight in a 4°C refrigerator, and serum was collected by centrifugation. The following day, BNP, CK-MB, and cTnI in the serum of mice were detected using ELISA kits (ml037594, ml037723, and ml001932; Mlbio, Shanghai, China), according to the manufacturer’s instructions.

### 2.17 Tissue processing and histopathology staining

Mouse heart tissues were fixed with 4% paraformaldehyde, embedded in paraffin, and sectioned to 5-μm thickness. H&E staining (G1120, Solarbio, China) was used to observe structural characteristics, and Masson’s trichrome staining (DC0033, Leagene Biotechnology, Beijing, China) was used to assess the degree of fibrosis in cardiac tissue. TUNEL staining (B0068, Lablead Biotech, China) was used to evaluate cardiomyocyte apoptosis. After staining, the slides were sealed with neutral resin, and images were collected using a Leica microscope.

To detect cardiac lipid accumulation, cardiac tissue was embedded in OCT medium, and 8-μm thick frozen sections were collected. The sections were stained with BODIPY 493/503 (4 μM), and images were obtained using fluorescence microscopy.

### 2.18 Detection of serum glucose, TG, TC, and LDL-C

Blood was collected from the orbital sinus of mice, and the serum was collected by centrifugation after keeping it in the refrigerator at 4°C overnight. The following day, mouse serum blood glucose, triglyceride (TG), total cholesterol (TC), and low-density lipoprotein cholesterol (LDL-C) levels were detected using a blood glucose content assay (BC2495, Solarbio), a TG assay kit (A110-1-1, Nanjing Jiancheng Bioengineering, China), a TC assay kit (A111-1-1, Nanjing Jiancheng Bioengineering, China), and an LDL-C assay kit (A113-1-1, Nanjing Jiancheng Bioengineering, China), respectively. All experiments were performed according to the manufacturer’s instructions.

### 2.19 Statistical analysis

All experiments were performed with three or more independent replicates. All data are presented as the mean ± SD. GraphPad Prism software (Version 10, GraphPad Software Inc., CA, United States) was used for statistical analyses. The two-tailed Student’s t-test was used to determine the differences between the two groups. One-way ANOVA followed by Tukey’s *post hoc* test was used to determine the differences among multiple groups. P < 0.05 was considered statistically significant.

## 3 Results

### 3.1 A-MG increases viability and inhibits apoptosis in HG/F-induced H9C2 cells

**FIGURE 1:**
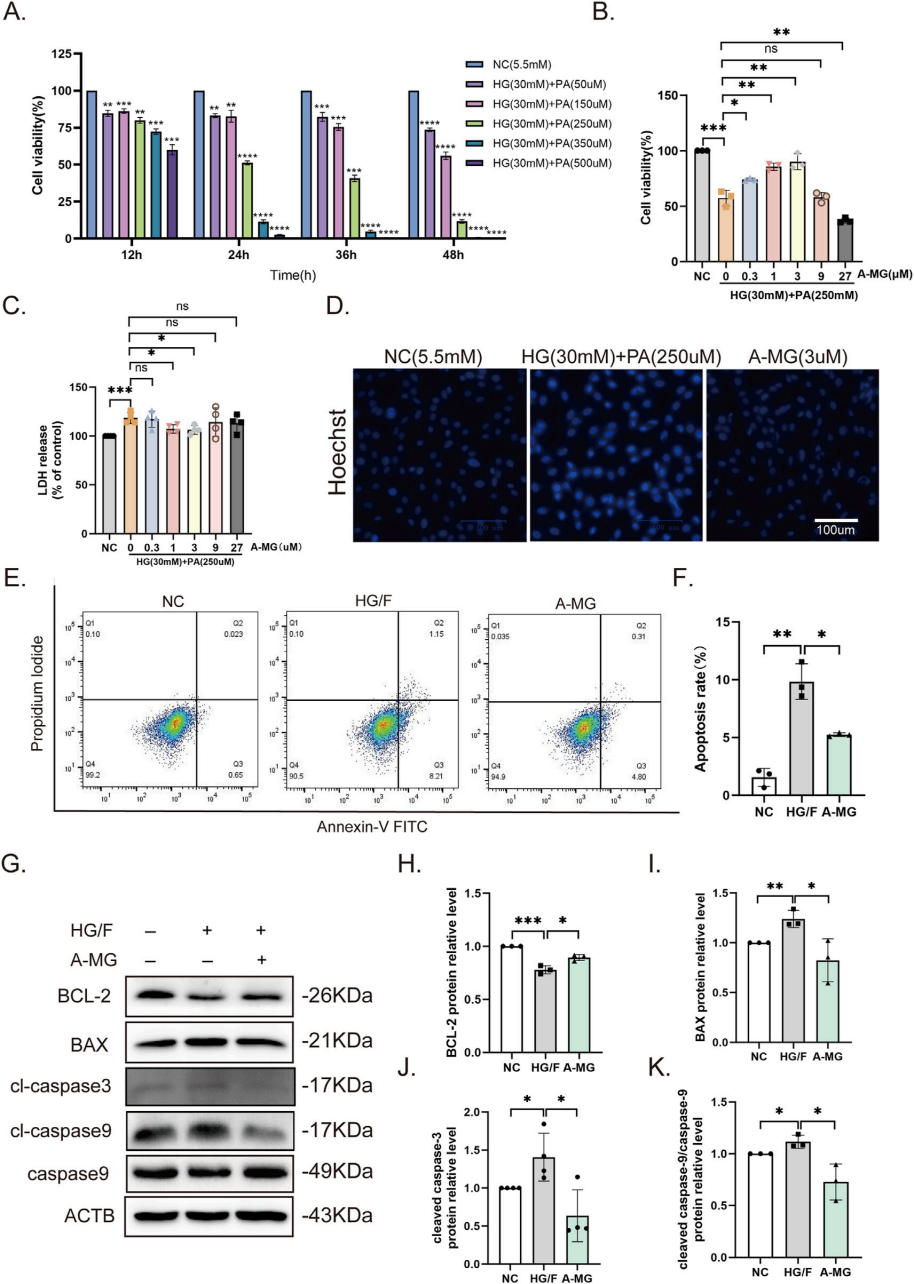
A-MG sustains viability and inhibits apoptosis in HG/F-induced H9C2 cells. **(A)** Results of CCK8 assays evaluating the viability of H9C2 cells exposed to high glucose and different concentrations of PA. **(B)** Results of CCK8 assays in HG/F-induced H9C2 cells treated with different concentrations of A-MG. **(C)** LDH release assay results. **(D)** Assessment of apoptotic damage in H9C2 cells by Hoechst 33258 staining. **(E, F)** Flow cytometry analysis of apoptosis in Annexin V/FITC-PI-labeled H9C2 cells. **(G–K)** Western blot analysis of BCL-2, BAX, cleaved caspase-3, cleaved caspase-9, and caspase-9 protein expressions in the NC, HG/F, and A-MG groups. The results represent at least three independent experiments; data are presented as the mean ± SD (n ≥ 3). ^*^p < 0.05, ^**^p < 0.01, ^***^p < 0.001, and ^****^p < 0.0001.

To establish an *in vitro* model of DCM-related cardiomyocyte damage, H9C2 cells were incubated with medium containing high glucose (HG, 30 mM) and different concentrations of PA for 12, 24, 36, or 48 h (Ma et al., 2021; Wu et al., 2022a). The results of CCK8 assays showed that cell viability decreased by 50% after 24-h treatment with HG and 250 μM PA. These cell culture parameters were then selected as a model condition for DCM-related myocardial injury (Figure 1A). To explore A-MG’s effect on the abovementioned myocardial injury model *in vitro*, HG/F-induced H9C2 cells were pretreated with A-MG at 0, 0.3, 1, 3, 9, or 27 μM. CCK8 assay results showed that A-MG at concentrations of 1 μM and 3 μM significantly improved cell viability (Figure 1B) and decreased LDH release, respectively (Figure 1C). Subsequently, Hoechst 33258 staining, flow cytometry, and Western blotting were used to detect apoptosis. In the HG/F model group, apoptosis was evidenced by dense, bright blue nuclei, reduced levels of the anti-apoptotic protein BCL-2, increased levels of pro-apoptotic proteins BAX and cleaved caspase-3, and an elevated cleaved caspase-9/caspase-9 ratio. After the addition of A-MG (3 μM), cell and nuclear morphologies were largely preserved (Figure 1D), the apoptosis rate decreased from 9.8% to 5.25% (Figures 1E,F), and BCL-2 expression was increased by 1.14-fold, while BAX, cleaved caspase-3, and the cleaved caspase-9/caspase-9 ratio were reduced by 33%, 54%, and 34%, respectively (Figures 1G–K). These results suggested that A-MG protects H9C2 cells against apoptosis induced by HG/F.

### 3.2 A-MG regulates Nrf2 signaling and increases the antioxidant capacity in HG/F-induced H9C2 cells

**FIGURE 2:**
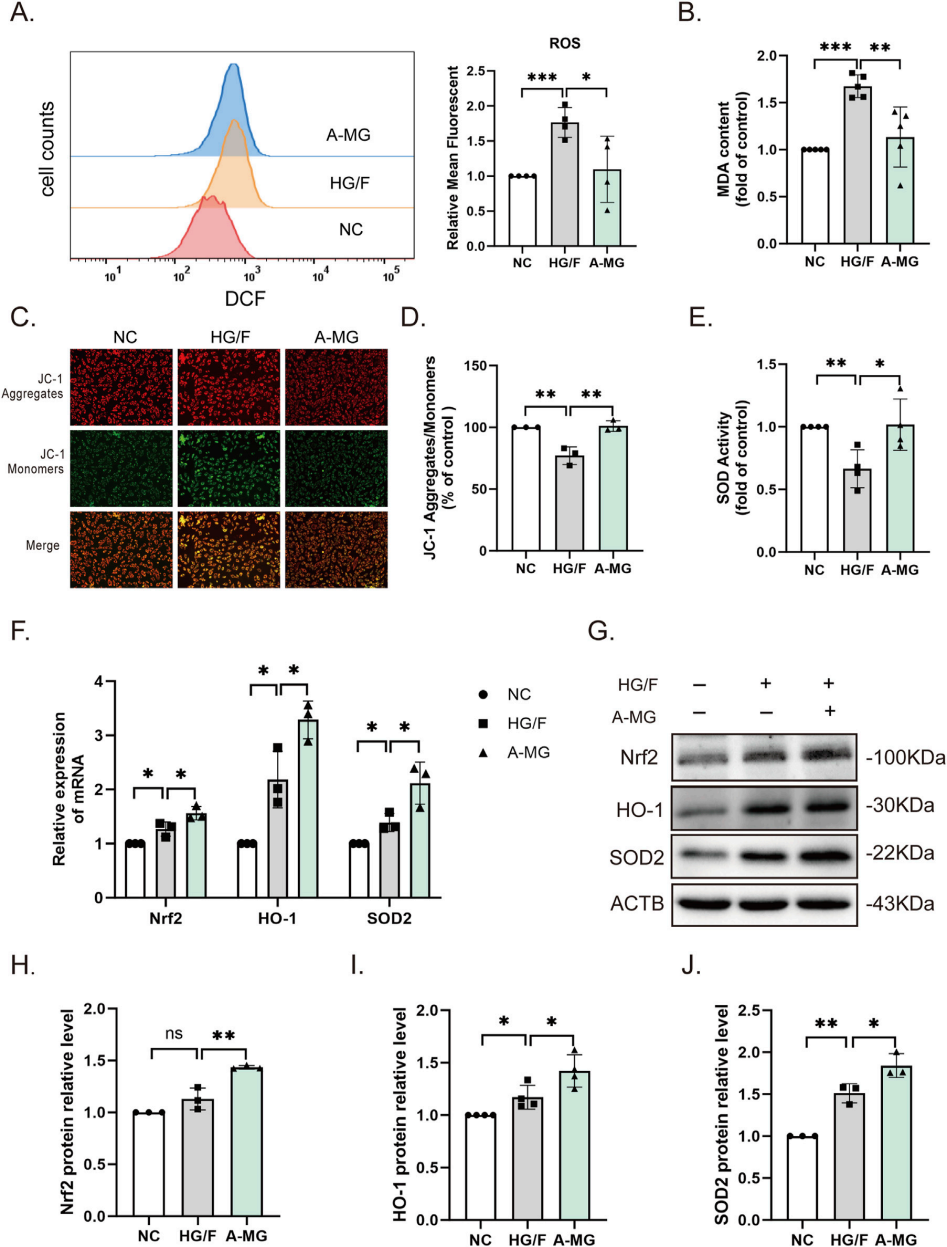
A-MG alleviates oxidative stress in HG/F-induced H9C2 cells. **(A)** Flow cytometry analysis of intracellular ROS generation in DCFH-DA-loaded H9C2 cells. **(B)** Quantification of MDA levels; data were normalized to the control group. **(C, D)** Representative images of JC-1-stained H9C2 cells **(C)** and corresponding quantitative analysis **(D)**. **(E)** SOD activity measurements in H9C2 cells. **(F)** RT-qPCR detection of Nrf2, HO-1, and SOD2 mRNA levels. **(G–J)** Western blot images and corresponding analysis of Nrf2, HO-1, and SOD2 expression in H9C2 cells. The results represent at least three independent experiments; data are presented as the mean ± SD (n ≥ 3). ^*^p < 0.05, ^**^p < 0.01, and ^***^p < 0.001.

To study the protective mechanism of A-MG against cardiomyocyte injury, we first used flow cytometry to detect intracellular ROS levels in H9C2 cells. ROS generation was significantly increased in the HG/F model group, and this change was markedly mitigated after A-MG treatment (Figure 2A). Lipid peroxidation levels were also increased in the HG/F model group and normalized after the addition of A-MG (Figure 2B). To evaluate whether excessive ROS production in HG/F-induced H9C2 cells leads to mitochondrial dysfunction (Szeto, 2017), we used JC-1 staining to detect changes in mitochondrial membrane potential. The results showed that HG/F conditioning led to mitochondrial membrane depolarization, evidenced by significantly lower red J-aggregate fluorescence to green monomer fluorescence, and this effect was largely reversed after A-MG exposure (Figures 2C,D). Nrf2, a transcription factor highly responsive to oxidative stress, activates cell’s antioxidant defenses, regulating the activity of antioxidant enzymes such as HO-1 and SOD1/2 (Gallorini et al., 2015; Tong and Zhou, 2017). We found that SOD activity was decreased in H9C2 cells in the HG/F model group but returned to normal levels after exposure to A-MG (Figure 2E). Subsequently, we detected mRNA and protein expression of Nrf2, HO-1, and SOD2. As shown in Figures 2F–J, exposure to HG/F for 24 h significantly activated the Nrf2/HO-1 pathway in H9C2 cells by enhancing mRNA and protein expression levels of Nrf2, HO-1, and SOD2. Moreover, 3 μM of A-MG further enhanced the Nrf2, HO-1, and SOD2 mRNA and protein expressions. These results suggest that A-MG can further promote the Nrf2/HO-1 pathway in H9C2 cells stimulated by HG/F.

### 3.3 A-MG alleviates lipid accumulation in HG/F-induced H9C2 cells through the FOXO1/CD36 pathway

**FIGURE 3:**
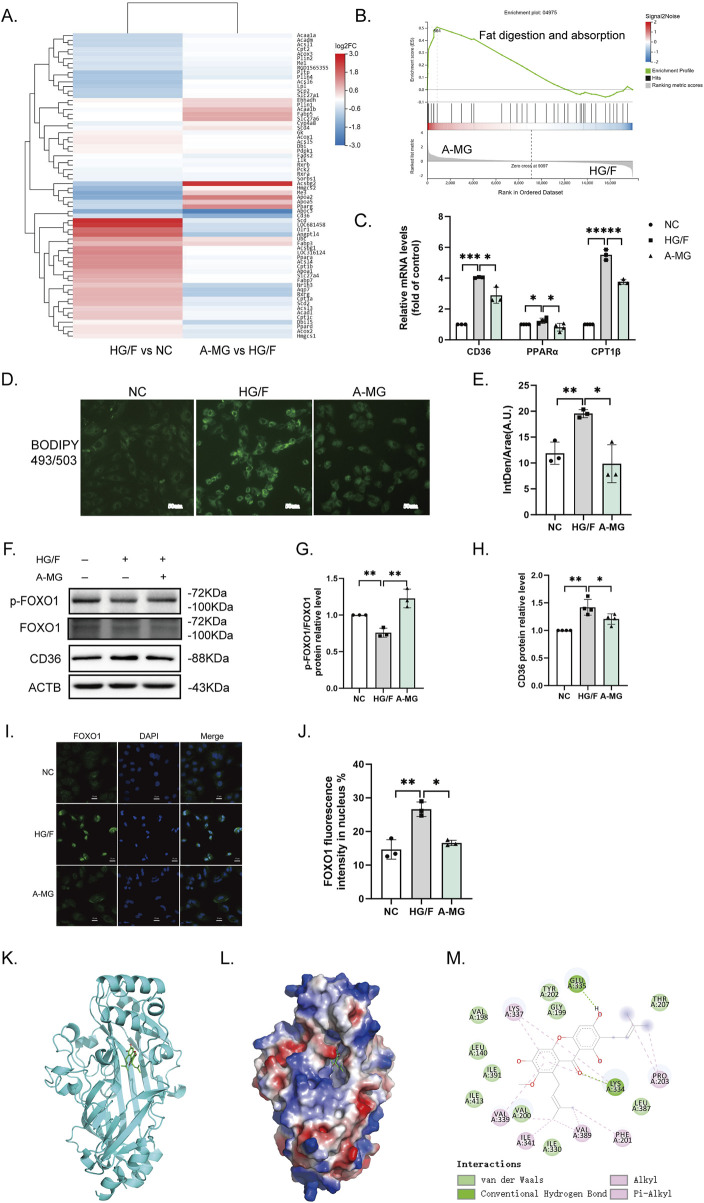
A-MG reduces lipid accumulation in HG/F-induced H9C2 cells. **(A)** Heatmap depicting A-MG-induced changes in gene expression influencing fat digestion and absorption. **(B)** GSEA gene enrichment map. **(C)** RT-qPCR analysis of CD36, PPARα, and CPT1β mRNA levels in H9C2 cells. **(D)** Representative images of intracellular lipid droplets in H9C2 cells stained with BODIPY 493/503. **(E)** Quantification of the mean fluorescence intensity of BODIPY 493/503; IntDen/Area (A.U.), integrated density/area (arbitrary unit). **(F–H)** Western blot analysis of p-FOXO1, FOXO1, and CD36 protein expressions in H9C2 cells. **(I)** Representative images of immunofluorescent staining of FOXO1 in H9C2 cells. **(J)** Quantification of the FOXO1 immunofluorescence signal in H9C2 cells. **(K–M)** Analysis of docking between A-MG and CD36 proteins. **(K)** 3D structure of CD36 bound with A-MG; the structure of CD36 is shown in cyan and A-MG is rendered in green. **(L)** Depiction of the electrostatic surface of the A-MG binding site on CD36. **(M)** Detail of the binding mode of A-MG on CD36. The results represent at least three independent experiments; data are expressed as the mean ± SD (n ≥ 3). ^*^p < 0.05, ^**^p < 0.01, and ^***^p < 0.001.

We next conducted RNA sequencing in H9C2 cells in the NC, HG/F, and A-MG groups. KEGG-based analysis of the transcriptome data indicated enrichment of differentially expressed genes (DEGs) in fat digestion and absorption processes, as summarized in the heatmap shown in Figure 3A. Consistent with these findings, GSEA results showed that the DEGs involved in metabolic pathways related to fat digestion and absorption were enriched in the HG/F and A-MG groups (Figure 3B). Subsequently, RT-qPCR assays revealed significant upregulation of CD36, peroxisome proliferator-activated receptor alpha (PPARα), and carnitine palmitoyltransferase 1 (CPT1β) in HG/F-induced H9C2 cells, which was reversed upon the addition of A-MG (Figure 3C).

We next employed BODIPY 493/503 staining to detect lipid droplet accumulation in H9C2 cells. We found that the accumulation of lipid droplets was significantly increased after HG/F induction and that A-MG treatment attenuated this effect (Figures 3D,E). Then, we detected phosphorylated FOXO1 (p-FOXO1) and CD36 levels by Western blotting and nuclear translocation of FOXO1 by immunofluorescence. The results showed that p-FOXO1 expression was decreased, while CD36 expression was markedly elevated in the HG/F group. After A-MG treatment, FOXO1 phosphorylation increased, whereas CD36 expression decreased significantly (Figures 3F–H). Immunofluorescence staining showed that approximately 14% of FOXO1 contents remained in the nucleus in the NC group, increasing to 27% after HG/F induction and decreasing to 17% after A-MG treatment (Figures 3I,J). These findings showed that A-MG can inhibit the nuclear translocation of FOXO1 and lower the expression of CD36.

Subsequently, we used molecular docking methods to evaluate the potential interaction between A-MG and CD36. Results showed that A-MG exhibits a strong affinity for CD36 (<−7.3 kcal/mol), with good docking performance (Figures 3K-M). Specifically, hydrogen bonds were predicted to mediate a strong interaction between A-MG and LYS-334 and GLU-335 at the active site of CD36, which is consistent with the hydrophobic binding of FAs to CD36. These results suggested that A-MG forms a structurally stable complex with CD36, potentially affecting its function.

### 3.4 A-MG attenuates β-oxidation of FAs in HG/F-induced H9C2 cells via FOXO1 regulation

**FIGURE 4:**
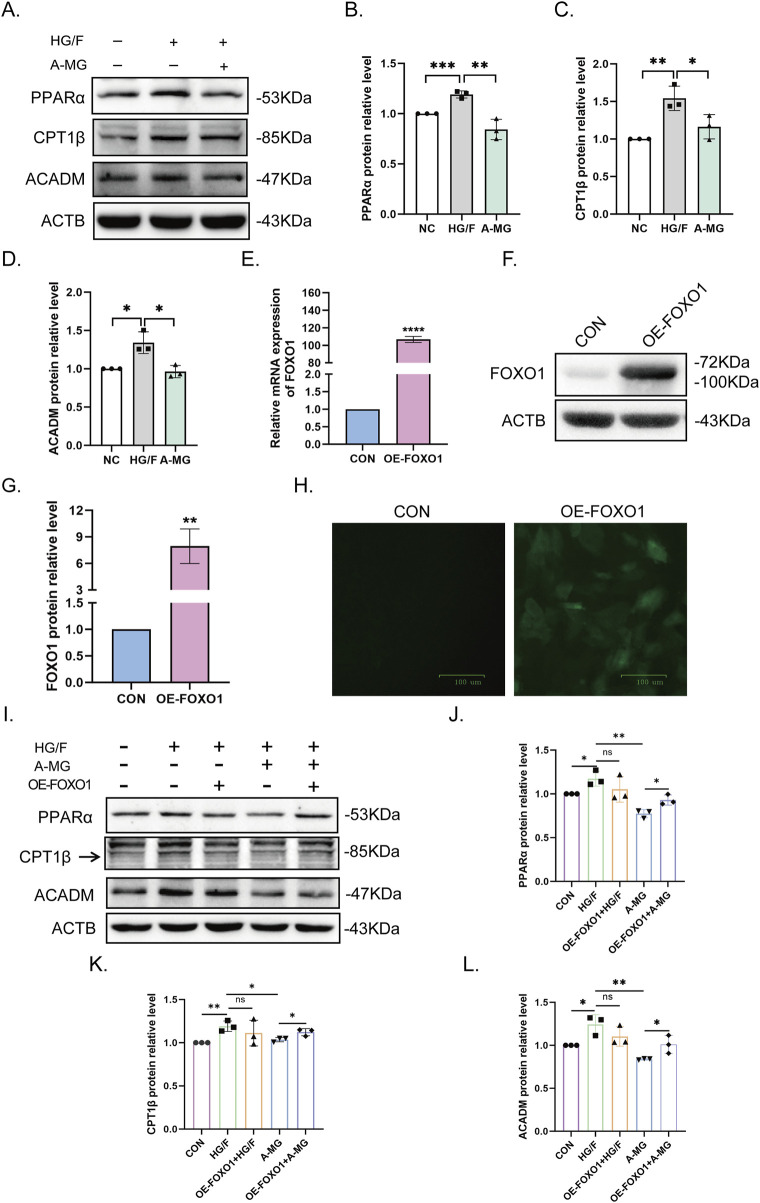
A-MG decreases fatty acid excessive β-oxidation in HG/F-induced H9C2 cells. **(A–D)** Western blot analysis of PPARα, CPT1β, and ACADM protein levels in H9C2 cells. **(E–G)** After lentiviral transduction, analysis of FOXO1 mRNA **(E)** and protein **(F)** expression in H9C2 cells; quantitative data are shown in **(G)**. **(H)** GFP fluorescence measurements in H9C2 cells transduced with FOXO-OE vectors. The data are presented as the mean ± SD (n = 3). ^*^p < 0.05, ^**^p < 0.01, ^***^p < 0.001, and ^****^p < 0.0001. **(I–L)** Western blot analysis of the effect of FOXO1 overexpression on PPARα, CPT1β, and ACADM expression in the five H9C2 cell groups. The results represent at least three independent experiments; data are expressed as the mean ± SD (n = 3). ^*^p < 0.05, ^**^p < 0.01.

Our RNA sequencing results revealed that A-MG not only affects the expression of FA absorption and transport-related genes but also regulates the β-oxidation of FAs. Therefore, we used Western blotting to study the expression of key molecules involved in this process, namely, PPARα, CPT1β, and medium-chain specific acyl-CoA dehydrogenase (ACADM). The results showed that their levels were notably elevated in the HG/F group and restored following exposure to A-MG (Figures 4A–D). These results pointed out that A-MG can inhibit excessive β-oxidation of FAs in HG/F-induced H9C2 cells.

To investigate whether FOXO1 contributes to excessive β-oxidation of FAs promoted by HG/F conditioning, we constructed a lentiviral GFP-based FOXO1 overexpression cell system (FOXO1-OE) and integrated it into H9C2 cells. After confirming successful transduction via RT-qPCR and Western blot assays (Figures 4E–H), we divided H9C2 cells into five groups, namely, NC, HG/F, OE-FOXO1+HG/F, A-MG, and OE-FOXO1+A-MG, and conducted Western blot assays to evaluate PPARα, CPT1β, and ACADM protein levels in each group. The results showed that the mitigating effect of A-MG on the downregulation of the three β-oxidation markers induced by HG/F conditioning was negated after the overexpression of FOXO1 (Figures 4I–L). These results indicated that A-MG inhibits the nuclear translocation of FOXO1, which consequently prevents excessive β-oxidation of FAs mediated by the PPARα/CPT1β pathway.

### 3.5 A-MG enhances antioxidant capacity and reduces HG/F-induced β-oxidation by activating AKT

**FIGURE 5:**
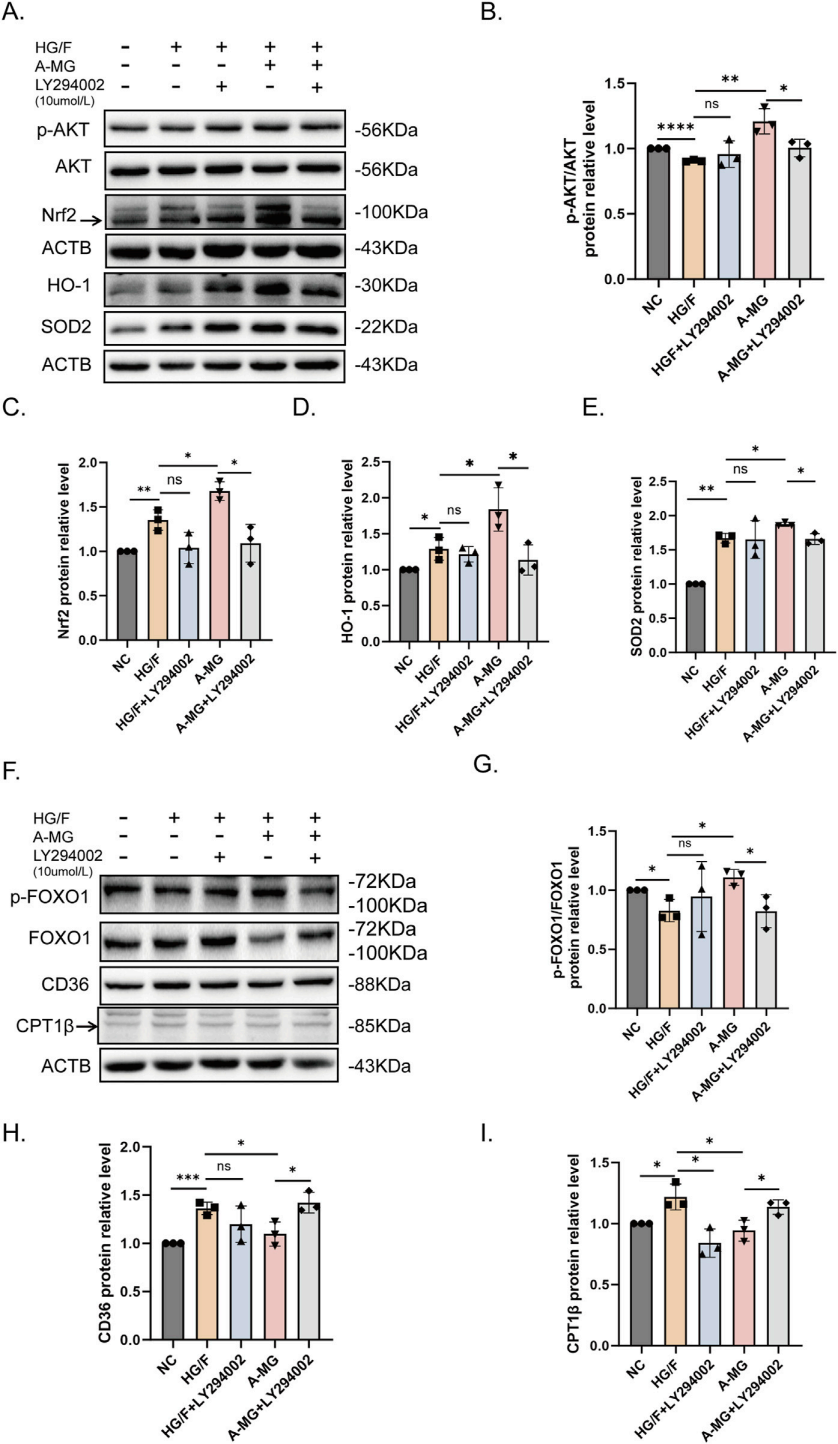
A-MG activates the AKT pathway. **(A–E)** Western blot analysis examining the effect of LY294002 on AKT, p-AKT, Nrf2, SOD2, and HO-1 protein expression in HG/F-induced H9C2 cells. **(F–I)** Western blot analysis examining the effect of LY294002 on FOXO1, p-FOXO1, CD36, and CPT1β expression in HG/F-induced H9C2 cells. The results represent at least three independent experiments; data are expressed as the mean ± SD (n = 3). ^*^p < 0.05, ^**^p < 0.01, ^***^p < 0.001, and ^****^p < 0.0001.

Our previous results showed that A-MG promotes AKT phosphorylation. To explore whether A-MG enhances antioxidant capacity and inhibits FA absorption by promoting AKT phosphorylation, we used LY294002, a PI3K/AKT inhibitor, to inhibit AKT activation. Western blot assays showed that exposure to 10 μM of LY294002 (Zhang et al., 2023) prevented A-MG-induced AKT phosphorylation and the expression of Nrf2, SOD2, and HO-1 (Figures 5A–E). Furthermore, Western blot assays showed that incubation with LY294002 reversed the stimulatory effect of A-MG on p-FOXO1 expression and its inhibitory effect on CD36 and CPT1β expression (Figures 5F–I). These findings indicated that A-MG enhances the antioxidant capacity of HG/F-induced H9C2 cells by upregulating the Nrf2/HO-1 signaling pathway and reducing excessive β-oxidation through CD36-mediated FA transport inhibition and AKT/FOXO1 signaling activation.

### 3.6 A-MG enhances cardiac systolic function and reduces myocardial injury in diabetic mice

**FIGURE 6:**
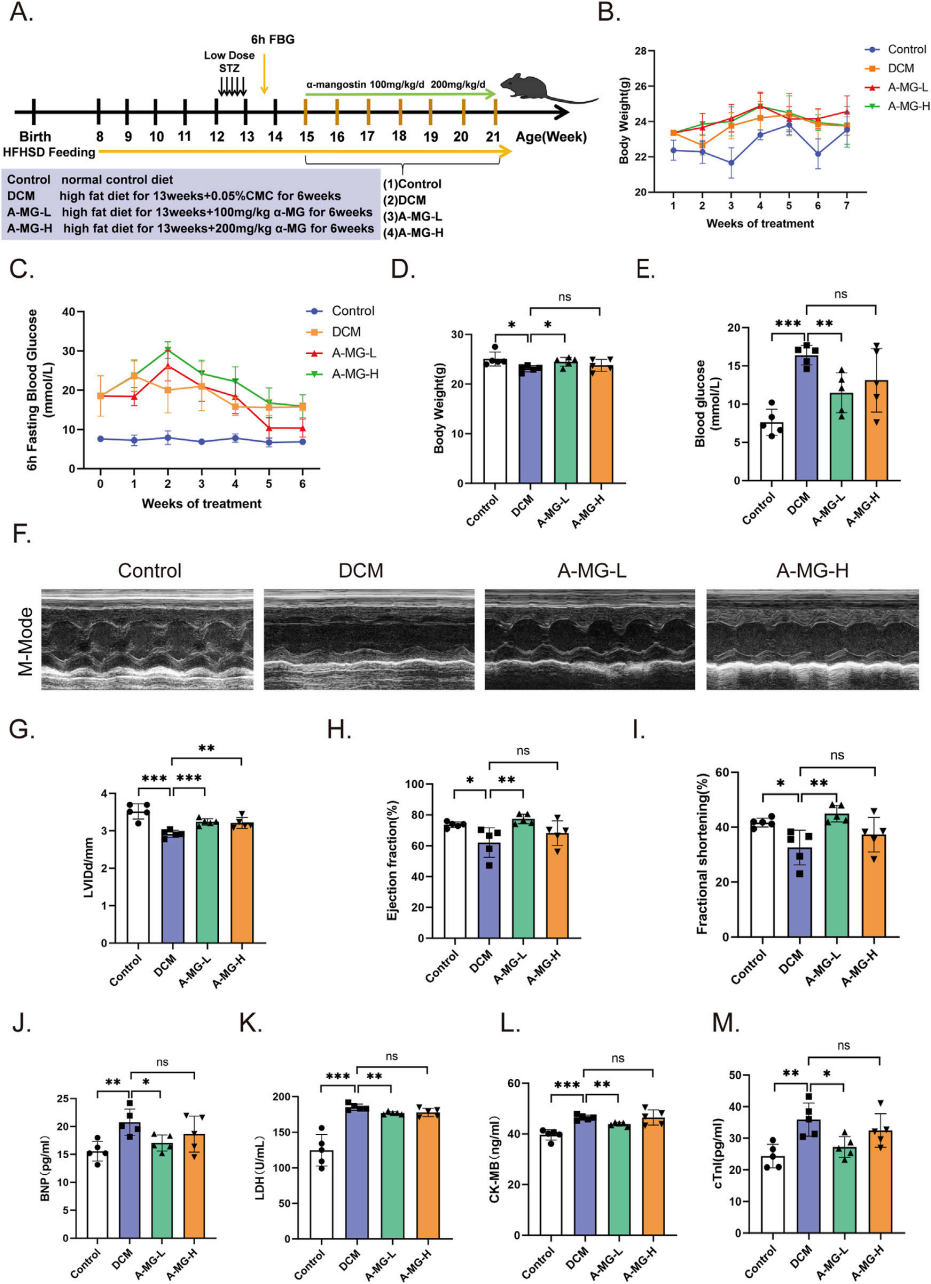
A-MG enhances cardiac systolic function and reduces myocardial injury in a mouse model of DCM. **(A)** Grouping and timeline of animal experimental protocols. **(B)** Body weight change curves recorded during the 6-week period of A-MG administration. **(C)** FBG curves recorded during the 6-week period of A-MG administration. **(D, E)** Body weight **(D)** and blood glucose **(E)** measurements recorded after the conclusion of the A-MG treatment. **(F)** Representative echocardiographic images of the M-mode for mice in each experimental group. **(G–I)** Effects of A-MG on LVIDd (mm) **(G)**, LVEF (%) **(H)**, and FS (%) **(I)**. **(J–M)** Detection of BNP, LDH, CK-MB, and cTnI levels in serum. Data are presented as the mean ± SD, n = 5. ^*^p < 0.05, ^**^p < 0.01, and ^***^p < 0.001.

To evaluate the cardioprotective actions of A-MG *in vivo*, a mouse model of DCM was established by feeding wild-type C57/6J mice an HFD combined with low-dose STZ injection. The T2D mice were then divided into a DCM model group (no A-MG), a low-dose A-MG group (receiving intragastric A-MG 100 mg/kg/d for 6 weeks), and a high-dose A-MG group (receiving intragastric A-MG 200 mg/kg/d for 6 weeks) (Figure 6A); the dose of A-MG was compared to that in the previous study (Soetikno et al., 2020). A separate group of mice receiving neither HFD nor STZ served as the control. During the A-MG treatment period, the weight of mice in the low-dose A-MG group increased gradually, while blood glucose values, determined after a 6-h fasting period, showed fluctuations and an overall decrease (Figures 6B,C). Body weight decreased in the DCM model group, increased in the low-dose A-MG group, and showed no significant difference in the high-dose group compared to that in the control group (Figure 6D). After A-MG treatment, FBG levels were significantly reduced in the low-dose A-MG group and showed no differences in the high-dose A-MG group compared to those in the DCM model group (Figure 6E). Detailed data are shown in Table 2.

**TABLE 2:** Effect of A-MG on body weight, blood glucose, and cardiac function in diabetic mice (mean ± SD, n = 5).

Variable	Control	DCM	A-MG-L	A-MG-H
Weight (g)	25.054 ± 1.406	23.1 ± 0.613*^1^	24.49 ± 0.901*^2^	23.778 ± 1.193
Glucose (mmol/L)	7.622 ± 1.711	16.405 ± 1.273***^1^	11.475 ± 2.608**^2^	13.106 ± 4.171
LVIDd (mm)	3.518 ± 0.203	2.919 ± 0.097***^1^	3.240 ± 0.086***^2^	3.210 ± 0.143**^3^
LVEF (%)	73.598 ± 1.833	62.078 ± 10.488*^1^	77.401 ± 3.094**^2^	68.159 ± 7.969
FS (%)	41.673 ± 1.617	32.583 ± 6.304*^1^	44.940 ± 2.992**^2^	37.263 ± 6.286
BNP (pg/mL)	15.569 ± 1.758	20.776 ± 2.343**^1^	17.052 ± 1.430*^2^	18.638 ± 3.220
LDH (U/mL)	124.409 ± 21.979	185.204 ± 4.462***^1^	176.720 ± 1.931**^2^	177.792 ± 5.713
CK-MB (ng/mL)	39.622 ± 2.041	46.424 ± 1.002***^1^	43.865 ± 0.695**^2^	46.478 ± 2.983
CTnI (pg/mL)	24.37 ± 3.71	35.89 ± 5.24**^2^	27.21 ± 3.32*^1^	32.46 ± 5.3

Notes: LVEF, left ventricular ejection fraction; FS, fractional shortening; LVIDd, left ventricular internal end-diastolic diameter. *^1^p < 0.05, **^1^p < 0.01, and ***^1^p < 0.001 DCM vs. Control; *^2^p < 0.05, **^2^p < 0.01, and *** ^2^p < 0.001 A-MG-L vs. DCM; **^3^p < 0.01 A-MG-H vs. DCM.

Echocardiographic data demonstrated decreased cardiac systolic function in DCM model mice compared with control mice (Figure 6F). Notably, LVIDd, LVEF, and FS were all significantly improved after low-dose A-MG treatment. In turn, increased LVIDd and no significant difference in LVEF and FS were noted in mice in the high-dose A-MG group (Figures 6G–I). Furthermore, increased serum levels of BNP, LDH, CK-MB, and cTnI, indicative of cardiac damage, were observed in the DCM model group. These changes were significantly attenuated in the low-dose A-MG group; the trends of BNP, LDH, CK-MB, and cTnI in the high-dose group were decreased, but the difference was not significant (Figures 6J–M). Detailed data are shown in Table 2.

### 3.7 A-MG attenuates myocardial fibrosis and apoptosis in DCM mice

**FIGURE 7:**
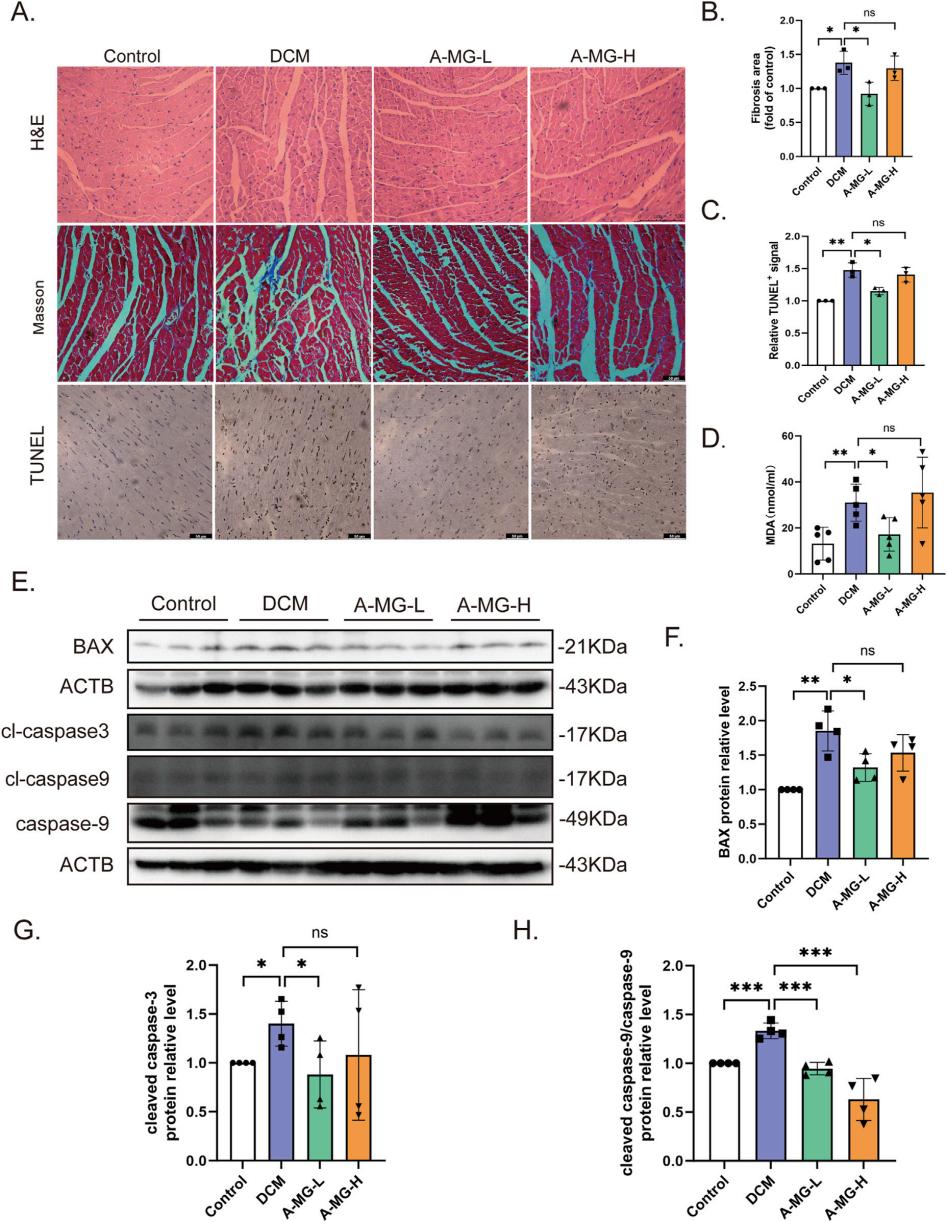
A-MG attenuates myocardial fibrosis and apoptosis in DCM mice. **(A)** Representative images of H&E staining, Masson’s trichrome staining, and TUNEL staining of myocardial tissue. **(B, C)** Quantitative analysis of myocardial fibrosis **(B)** and TUNEL-positive signal **(C)** in cardiac tissue. **(D)** Quantification of serum MDA levels in experimental mice. **(E–H)** Western blot analysis of BAX, cleaved caspase-3, cleaved caspase-9, and caspase-9 expression in cardiac tissues. Data are presented as the mean ± SD. In **(A–C)**, n = 3; in **(D)**, n = 5; in **(E–H)**, n = 4. ^*^p < 0.05, ^**^p < 0.01, and ^***^p < 0.001.

H&E staining showed that the arrangement and morphology of cardiomyocytes in the DCM model group of mice were disordered, irregularly shaped, and evidenced local muscle fiber rupture compared to those in the control group. Myocardial cells in the A-MG low-dose group were more uniform and regular and presented much less extensive fiber rupture than those in the DCM group. In contrast, in the high-dose A-MG group, these protective effects were less obvious. Masson’s trichrome staining showed that low-dose A-MG treatment significantly reduced myocardial fibrosis and that there was no significant difference in the high-dose group. We also detected apoptosis in myocardial tissue by TUNEL assays. Compared with that in the DCM model group, a significant reduction in the apoptosis rate was observed in the low-dose A-MG group but not in the high-dose A-MG group (Figures 7A–C). We further assessed serum levels of MDA, a surrogate marker of lipid peroxidation resulting from oxidative stress, in the different mouse groups. Serum MDA levels were significantly increased in the DCM model group compared to those in control mice. After treatment with A-MG, MDA levels tended to normalize in the low-dose group but remained elevated in the high-dose group (Figure 7D). Finally, Western blot analysis confirmed that various apoptosis-related markers (BAX, cleaved caspase-3, and the cleaved caspase-9/caspase-9 protein ratio) were elevated in the myocardium of DCM group mice but reduced instead after low-dose A-MG treatment (Figures 7E–H).

### 3.8 A-MG attenuates lipid accumulation and oxidative stress in DCM mice

**FIGURE 8:**
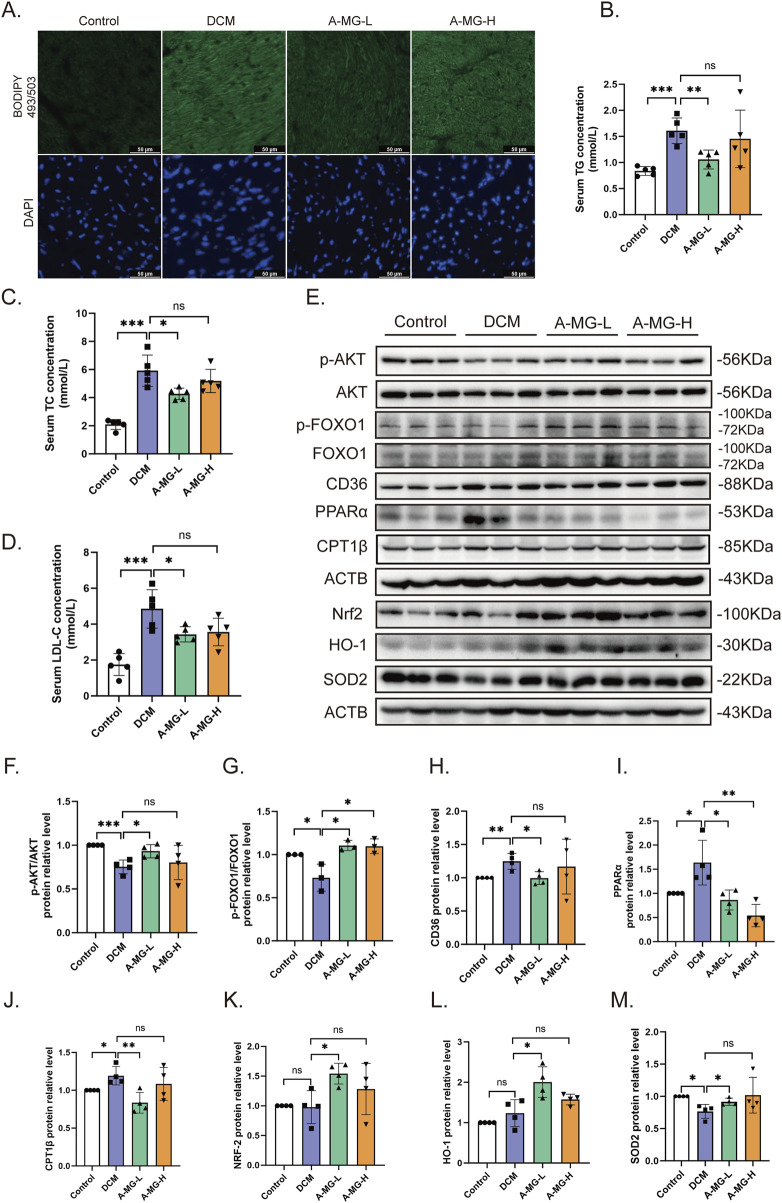
A-MG attenuates lipid accumulation and oxidative stress in the cardiac tissue of DCM mice. **(A)** Representative images of myocardial BODIPY 493/503 staining. **(B–D)** Analysis of serum TG, TC, and LDL-C levels. **(E–M)** Western blot analysis of p-AKT, AKT, p-FOXO1, FOXO1, CD36, PPARα, CPT1β, Nrf2, HO-1, and SOD2 in heart tissues. Data are presented as the mean ± SD. In **(B–D)**, n = 5; in **(F, H–M)**, n = 4; in **(G)**, n = 3. ^*^p < 0.05, ^**^p < 0.01, and ^***^p < 0.001.

Myocardial lipid accumulation is a hallmark feature of DCM (Nakamura and Sadoshima, 2020). To assess whether A-MG counteracts this phenomenon, we stained cardiac tissue samples with BODIPY 493/503 and monitored serum TG, TC, and LDL-C levels in DCM mice. The BODIPY fluorescence signal and serum TG, TC, and LDL-C levels were significantly increased in DCM model mice compared to those in control mice. Notably, low doses of A-MG were more effective in restoring these indicators than high doses (Figures 8A–D; Table 3). These findings indicated that A-MG ameliorates cardiac lipotoxicity resulting from DCM.

**TABLE 3:** Effect of A-MG on serum lipid levels of diabetic mice (mean ± SD, n = 5).

Variable	Control	DCM	A-MG-L	A-MG-H
TG (mmol/L)	0.837 ± 0.084	1.609 ± 0.245***^1^	1.508 ± 0.182**^2^	1.454 ± 0.551
TC (mmol/L)	2.092 ± 0.314	5.92 ± 0.989***^1^	4.272 ± 0.366*^2^	5.188 ± 0.736
LDL (mmol/L)	1.748 ± 0.613	4.850 ± 1.072***^1^	3.434 ± 0.424*^2^	3.562 ± 0.765

Notes: *** ^1^p < 0.001 DCM vs. Control; *^2^p < 0.05 and **^2^p < 0.01 A-MG-L vs. DCM.

Subsequently, we used Western blotting to measure the expression levels of proteins associated with lipid accumulation and FA oxidation pathways in the myocardium of control and DCM mice (Figures 8E–J). Compared to those of control mice, AKT and FOXO1 phosphorylation levels were reduced in DCM model mice but returned to normal levels after treatment with low-dose A-MG. In turn, compared to those in control mice, the expression levels of CD36, PPARα, and CPT1β were increased in the heart tissues of DCM model mice, and this effect was attenuated in low-dose A-MG-treated mice. These findings imply that A-MG safeguards against DCM by effectively reducing lipid accumulation and curbing excessive β-oxidation in cardiac cells. Moreover, pointing to a protective effect against oxidative stress in the setting of DCM, Western blot assay results indicated that A-MG treatment restored the cardiac expression of Nrf2, HO-1, and SOD2 in DCM mice (Figures 8E,K–M).

## 4 Discussion

**FIGURE 9:**
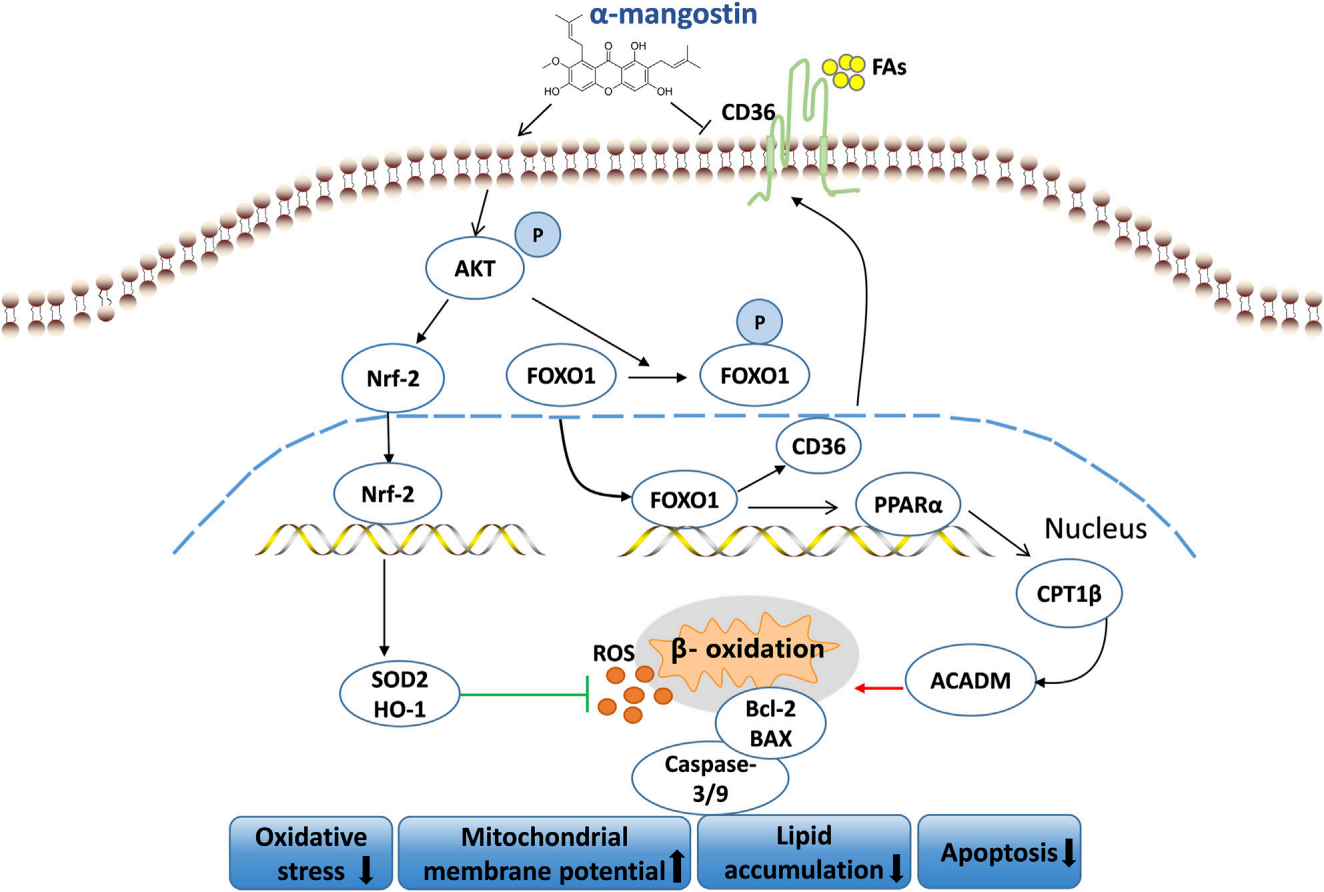
Mechanism of action of A-MG in DCM. A-MG effectively boosts mitochondrial membrane potential, mitigates oxidative stress, lipid accumulation, and apoptosis, and alleviates DCM.

This study proved that A-MG has a protective effect in DCM. On one hand, we showed that A-MG inhibits CD36-dependent transport of FAs and reduces lipid accumulation in cardiomyocytes by regulating the AKT/FOXO1 axis. On the other hand, we demonstrated that A-MG inhibits excessive β-oxidation of FAs mediated by the PPARα/CPT1β pathway by inactivating FOXO1. Furthermore, we showed that A-MG triggers the activation of the AKT/Nrf2/HO-1 signaling pathway, thereby boosting the myocardial antioxidant capacity. This evidence suggests that A-MG is a potential therapeutic agent for the treatment of DCM (Figure 9).

Based on autopsy findings, Rubler et al. (1972) defined DCM as a unique abnormality of ventricular structure and function in DM patients independent of hypertension, coronary heart disease, or other underlying cardiac causes. Over recent years, numerous diverse mechanisms have been put forward as having a role in this clinical condition. These mechanisms mainly encompass oxidative stress, fibrotic processes, cardiomyocyte death, mitochondrial malfunction, and changes in myocardial energy metabolism (Parim et al., 2019; Russo and Frangogiannis, 2016; Marwick et al., 2018). However, the precise etiology of DCM remains elusive, and so far, no specific treatment for DCM has been advanced.

Therefore, in this study, we addressed the pathogenic mechanisms of DCM and the therapeutic potential of A-MG using cell-based and *in vivo* models. *In vitro*, DCM was modeled by preconditioning H9C2 cells in the culture medium containing high glucose (30 mM) and 250 μM PA (HG/F). The success of this conditioning protocol was evidenced by decreased cell viability, augmented LDH release, excessive ROS and MDA production, intracellular lipid accumulation, reduced mitochondrial membrane potential, and apoptosis induction. *In vivo*, DCM was modeled in C57BL/6J wild-type male mice fed an HFD during 4 weeks, followed by a series of STZ injections over five consecutive days. Successful establishment of the DCM mouse model was evidenced by impaired cardiac function, structural abnormalities of cardiac tissue, increased levels of serum biomarkers of dyslipidemia and cardiac dysfunction, and cellular and molecular findings indicative of oxidative stress, excessive β-oxidation, and apoptosis.

A-MG is the principal active component in the fruit of Garcinia mangostana L. (Clusiaceae), a tropical tree native to Southeast Asian countries. A-MG exerts potent antioxidant effects in several disease settings, including heart disease. Studies have found that A-MG plays a protective role in ischemic heart disease by suppressing oxidative stress and reducing cardiomyocyte apoptosis (Fang et al., 2018), protects the heart from toxic damage caused by doxorubicin (Eisvand et al., 2022), and mediates the significant reduction in systolic and diastolic blood pressure in spontaneously hypertensive rats by downregulating angiotensin II expression (Xu et al., 2024b). Our study further expands evidence on the cardioprotective actions of A-MG by highlighting its ability to reduce oxidative stress, attenuate cardiomyocyte apoptosis, and enhance heart function related to DCM.

Abnormal glycolipid metabolism is the basic pathological feature of DCM, and mitochondrial damage associated with hyperglycemia and palmitate-induced cardiac lipotoxicity are the most well-known causes of elevated ROS levels in DCM (Ke et al., 2023). When large amounts of FAs enter the cytoplasm, their storage rate exceeds their oxidation rate, causing intracellular lipid accumulation and subsequent lipotoxicity that damages cells. CD36 is of great significance in the process of FA uptake. Long-chain FAs reach the transport tunnel of CD36 through a hydrophobic binding pocket on its surface (Kuda et al., 2013). Studies have shown that in diabetic db/db mouse cardiomyocytes, overexpression of CD36 leads to increased uptake of long-chain FAs and accumulation of TG, inducing cardiac lipotoxicity and systolic dysfunction (D'Souza et al., 2016). In this study, RNA sequencing results showed that CD36 expression is increased after HG/F induction, and the ability of A-MG to effectively reduce CD36 expression and lipid accumulation was confirmed by both *in vitro* and *in vivo* experiments. Furthermore, our *in silico* docking studies showed that A-MG docks with high affinity to the hydrophobic pocket of CD36. Specifically, this analysis revealed that A-MG can form strong hydrogen bond interactions with LYS-334 and GLU-335 at the site of FA entry in CD36. A conserved salt bridge between LYS-334 and GLU-335 is a key feature of the protein’s architecture. Additionally, LYS-332 plays a crucial role by enhancing the hydrophobicity of the binding pocket, which facilitates the proper positioning of FAs via interaction with the carboxyl groups of LYS-334 (Wang et al., 2019). Therefore, A-MG may affect CD36 transport function by competitively interacting with FAs, thereby reducing intracellular lipid accumulation to mitigate lipotoxicity, oxidative stress, and apoptosis of cardiac cells.

In cardiomyocytes, approximately 60%–70% of the energy is generated through the oxidation of long-chain FAs, with the remainder originating mainly from glucose metabolism, such as glycolysis and glucose oxidation (Liedtke, 1981; Camici et al., 1989). Studies have shown that in diabetic states, glycolysis is reduced by ∼52% and glucose oxidation is reduced by ∼84%, with concomitantly and significantly increased FA uptake and oxidation of palmitate (a saturated FA) (Ke et al., 2023). The metabolic preference for FAs, which eventually leads to cellular damage arising from mitochondrial overactivation, is a significant factor contributing to lipid-induced cardiotoxicity in diabetic individuals. In DCM, mitochondrial function is impaired. When the energy supply shifts to the preferential utilization of FAs, a large amount of FAs flows into cardiomyocytes, leading to the activation of PPARα, a member of the nuclear hormone receptor superfamily that acts as a central regulator of FA metabolism by transcriptionally regulating the expression of genes involved in mitochondrial β oxidation (Glatz and Luiken, 2017). Studies have shown an association between increased FA oxidation and reduced heart efficiency in animal models of diabetes (Zhao et al., 2004; Kandula et al., 2016). In this study, RNA sequencing of H9C2 cells showed that the expression of enzymes for FA β-oxidation increased after HG/F induction. Crucial to the process of FA β-oxidation are CPT1, an integral outer mitochondrial membrane protein that acts as a rate-limiting enzyme for β-oxidation, and ACADM, the first enzyme to catalyze mitochondrial FA oxidation (Tanaka et al., 1992; Angelini et al., 2021). Further experiments verified that HG/F induction caused an increase in cellular lipid accumulation and the expression of the FA β-oxidation-related enzymes PPARα, CPT1β, and ACADM and that A-MG reversed these effects. Thus, reducing cardiac oxidative damage by lowering the β-oxidation of FAs was a key mechanism by which A-MG mitigated oxidative stress and attenuated cardiomyocyte apoptosis.

FOXO transcription factors are involved in the pathogenesis of DCM through various mechanisms related to cell metabolism, apoptosis, and oxidative stress. FOXO activity is regulated by various post-translational modifications, such as phosphorylation, acetylation, and ubiquitination. Studies have shown that chronic insulin stimulation can activate FOXO1 to induce the translation of the CD36 mRNA and further upregulate the expression of the CD36 protein (Chistiakov et al., 2017). In a type 1 diabetes model, impaired insulin signaling promoted cardiac oxidative stress by activating FOXO1 and the expression of PPARα in cardiomyocytes (Kyriazis et al., 2021). The results of this study indicate that A-MG inhibits the transcriptional regulation of CD36 by promoting FOXO1 phosphorylation, thereby reducing lipid accumulation in cardiomyocytes. A-MG inhibits PPARα/CPT1β-mediated excess β-oxidation of FAs by inhibiting FOXO1 nuclear translocation. Therefore, A-MG lowers lipid toxicity by downregulating CD36 expression through FOXO1 and inhibits PPARα/CPT1β-mediated excessive β-oxidation of FAs to reduce oxidative damage. Our experimental model results suggest a strong therapeutic potential for A-MG in modulating the uptake of FAs and regulating β-oxidation in DCM.

During oxidative stress, the balance between pro-oxidant and antioxidant reactions is disrupted, resulting in the production of excessive ROS that causes cell damage and apoptosis. Nrf2 plays a crucial role in the resistance of cells to oxidative damage (Sporn and Liby, 2012). The activation of the Nrf2/HO-1 signaling pathway represents a crucial step in the antioxidant defense mechanism of the body (Wang et al., 2022; Gu et al., 2017; Yang et al., 2024). In this study, HG/F-induced cardiomyocytes showed increased expression of CD36, which led to increased lipid accumulation, excessive β-oxidation of FAs, reduced mitochondrial membrane potential, and oxidative stress triggered by excessive ROS generation. Notably, we showed that A-MG supplementation can enhance myocardial antioxidant capacity and restrain oxidative stress by promoting an antioxidant response via the upregulation of Nrf2 and its target genes *SOD2* and *HO-1*.

The PI3K/AKT signaling pathway crucially regulates blood glucose and lipid metabolism, influencing various processes related to inflammation and apoptosis in cardiomyocytes (Zhong et al., 2024). In a healthy and properly functioning heart, the AKT/FOXO1 pathway plays a crucial protective role by promoting metabolic adaptation through the action of enzymes involved in glycolysis and lipolysis. Such a mechanism prevents lipotoxicity and ensures the survival of cardiomyocytes exposed to high ceramide levels (Nirala et al., 2013). FOXO1 is inhibited by AKT via phosphorylation at three distinct sites, namely, Tyr-24, Ser-256, and Ser-319 (Guo and Guo, 2017). Studies have shown that reduced AKT-dependent FOXO1 phosphorylation is associated with insulin resistance and apoptosis in DCM (Duan et al., 2018). Our studies showed that A-MG promotes AKT phosphorylation, which inhibits FOXO1 activation and sustains glucose utilization at the expense of β-oxidation of FAs to reduce oxidative stress-related apoptosis of cardiomyocytes under HG/F conditions *in vitro* and DCM modeling *in vivo*.

Experiments in our mouse model of DCM showed that low-dose A-MG treatment significantly reduced blood glucose levels, improved ventricular systolic function, and reduced serum levels of myocardial damage marker enzymes. Moreover, consistent with our findings in HG/F-induced H9C2 myoblasts, H&E staining, TUNEL assays, BAX and caspase 3/9 Western blotting, and Masson’s trichrome staining showed that treatment with A-MG alleviated myocardial damage, cardiomyocyte apoptosis, and myocardial fibrosis supplementation in DCM mice. Furthermore, we found that after 6 weeks of treatment, low-dose (100 mg/kg/d) A-MG treatment significantly reduced cardiac lipid accumulation and serum TG, TC, and LDL-C levels, suggesting that A-MG can effectively reduce cardiac lipotoxicity associated with DCM. Finally, Western blotting showed that low-dose A-MG treatment increased cardiac expression of antioxidant proteins (Nrf2, HO-1, and SOD2), attenuated FA uptake by decreasing FOXO1 activation and CD36 expression, and counteracted β-oxidation of FAs by downregulating PPARα and CPT1β expression. Therefore, the mechanisms operating at the organ and cellular levels were mutually validated.

## 5 Conclusion

In summary, our study demonstrated that A-MG relieves DCM pathology by blocking the uptake of FAs via CD36, lowering systemic hyperglycemia and lipid accumulation in cardiac tissue, preventing excessive β-oxidation of FAs and ensuing ROS production, increasing the antioxidant capacity of cardiomyocytes, and inhibiting cardiomyocyte apoptosis. Specifically, our data indicate that A-MG inhibits apoptosis and alleviates myocardial injury by reducing myocardial lipid accumulation through the AKT/FOXO1/CD36 signaling pathway, lowering excessive β-oxidation of myocardial FAs through the FOXO1/PPARα/CPT1β signaling pathway, and improving myocardial antioxidant capacity by enhancing AKT/Nrf2/HO-1 signaling. These findings indicate that A-MG holds promise as a viable approach for the treatment of DCM.

## Data Availability

The original contributions presented in the study are included in the article/Supplementary Material; further inquiries can be directed to the corresponding author.
