# Supplementary material for: α-Mangostin prevents diabetic cardiomyopathy by inhibiting oxidative damage and lipotoxicity through the AKT–FOXO1–CD36 pathway
Source: Front Pharmacol. 2025 Apr 17;16:1566311. doi: 10.3389/fphar.2025.1566311 (PMC12043880; doi:10.3389/fphar.2025.1566311)
Supplement: Supplementary file 1 [file DataSheet1.pdf]

α-Mangostin prevents diabetic cardiomyopathy by inhibiting oxidative damage and lipotoxicity through AKT-FOXO1-CD36 pathway

Xue Bai[1], Ziqian Zhang[1], Miao Zhang[1], Jiaojiao Xu[1], Keting Dong[1], Qian Du[1], Lei Chen[1], Ping Ma[1] and Jianhong Yang[1*]

[1]Medical School, University of Chinese Academy of Sciences, Beijing 100049, P.R. China

*Correspondence to: Professor Jianhong Yang, Medical School, University of Chinese Academy of Sciences, 19A Yuquan Road, Beijing 100049, P.R. China

E-mail: yangjh@ucas.edu.cn

Supplemental information

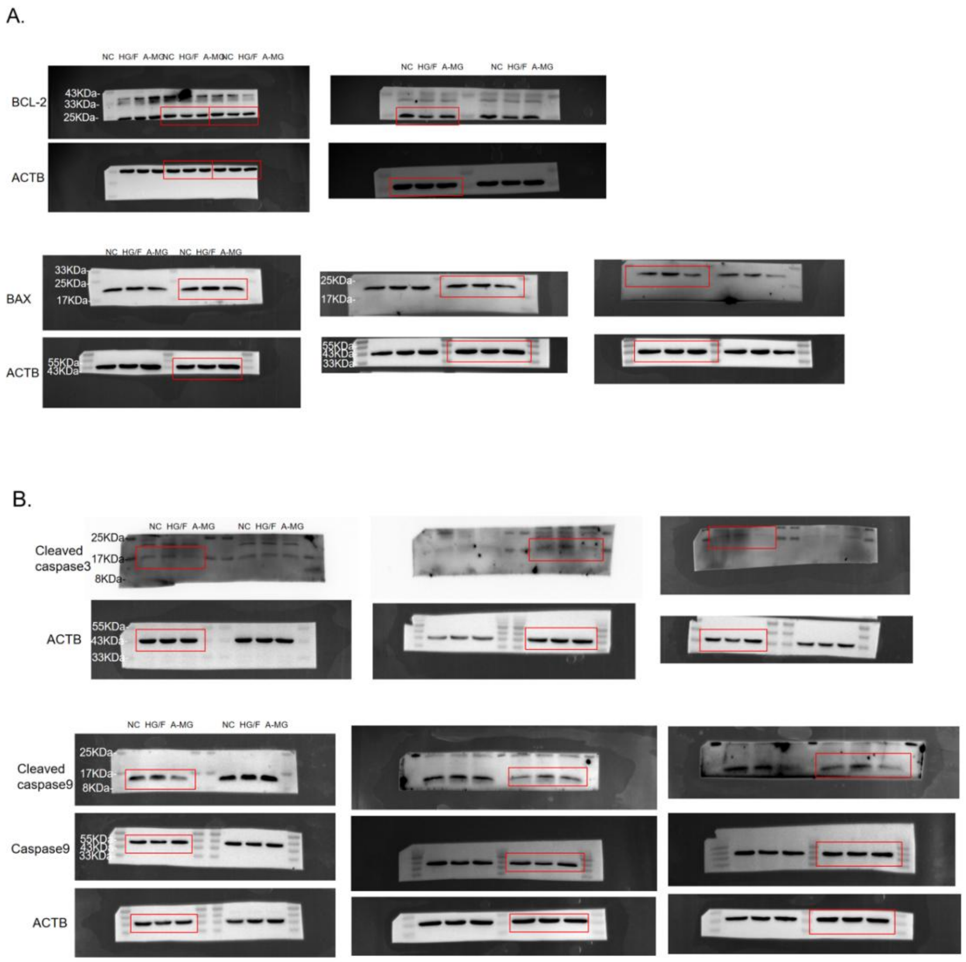

Figure S1 Western blot full size image of H9C2 cells. Figure **A-B** Shows the H9C2 cells were grouped into NC, HG/F, A-MG. The expression of apoptosis-related proteins BCL-2, BAX, Caspase3, Caspase9 were detected by Western blot.

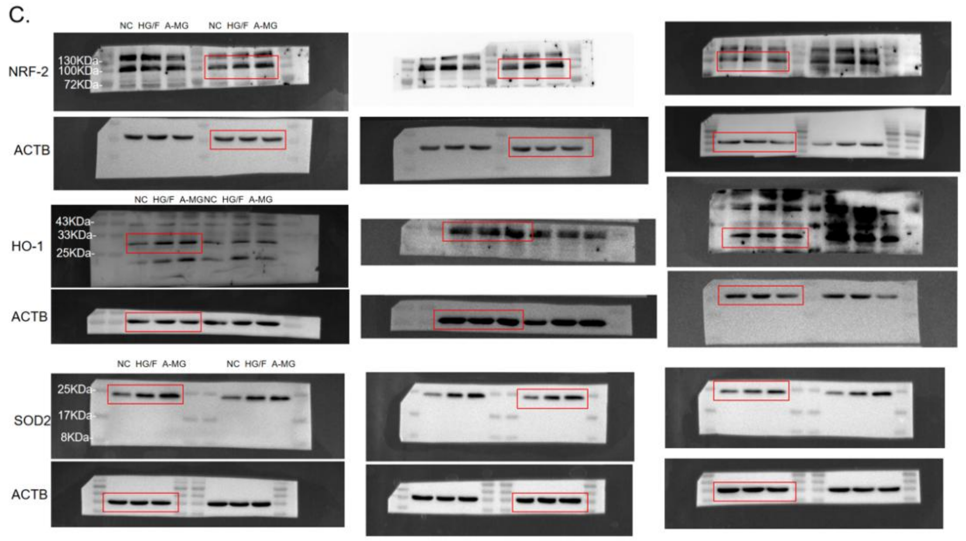

Figure S2 Western blot full size image of H9C2 cells. Figure **C** Shows the H9C2 cells were grouped into NC, HG/F, A-MG. Western blot was used to detect the expression of oxidative stress-related protein NRF-2, HO-1, SOD2.

D.

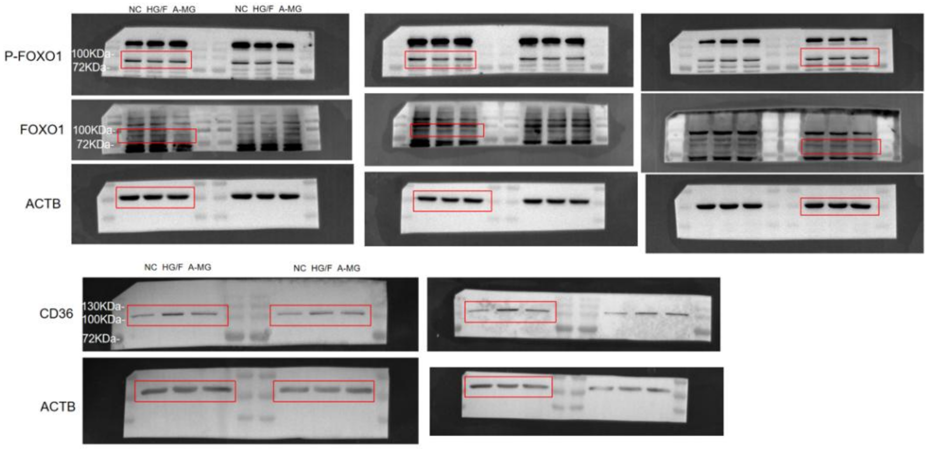

Figure S3 Western blot full size image of H9C2 cells. Figure **D** Shows the H9C2 cells were grouped into NC, HG/F, A-MG. Western blot analysis of FOXO1 and CD36 protein expression associated with fatty acid uptake.

E.

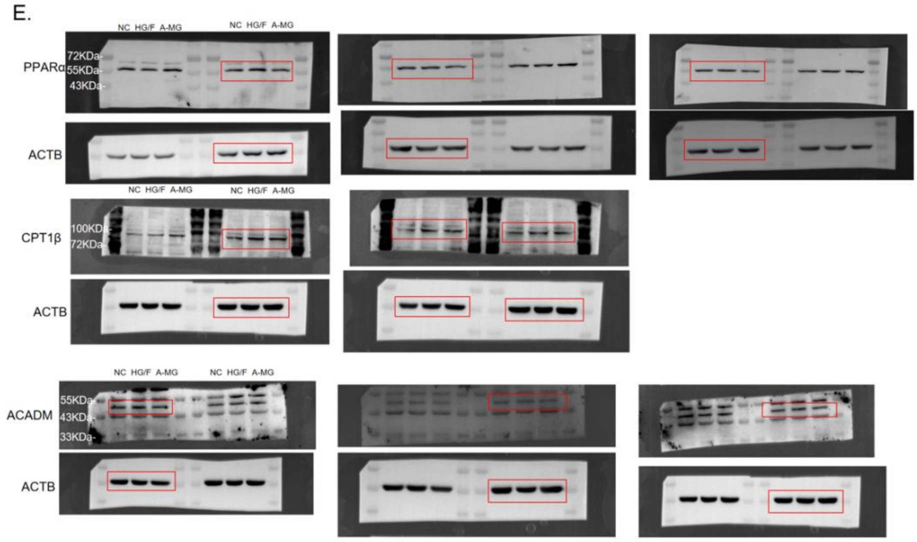

Figure S4 Western blot full size image of H9C2 cells. Figure **E** Shows the H9C2 cells were grouped into NC, HG/F, A-MG. Western blot analysis of PPARα, CPT1β, ACADM protein expression related to fatty acid β oxidation.

F.

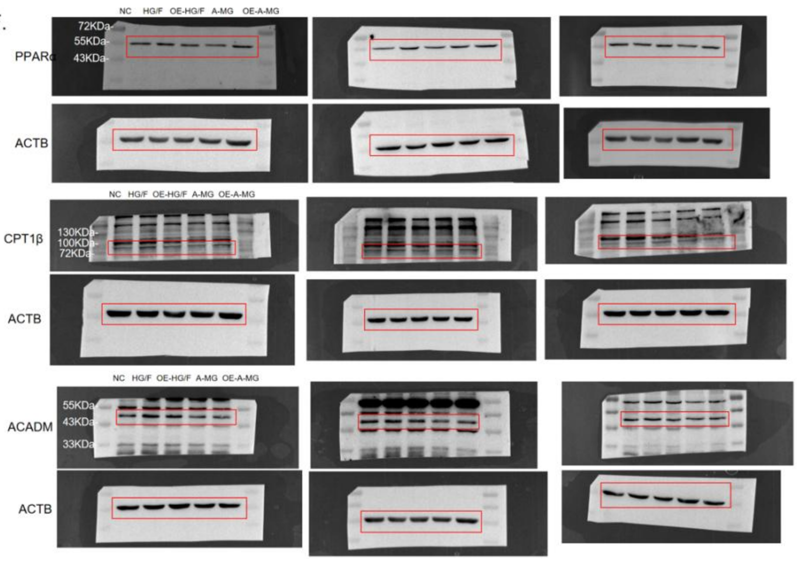

Figure S5 Western blot full size image of H9C2 cells. Figure **F** Shows that normal and overexpressing FOXO1 in H9C2 cells were grouped into NC, HG/F, OE+HG/F, A-MG, OE+A-MG. Western blot analysis of PPARα, CPT1β, and ACADM protein expression, where OE+HG/F=OE-FOXO1+HG/F, OE+A-MG=OE-FOXO1+A-MG.

G.

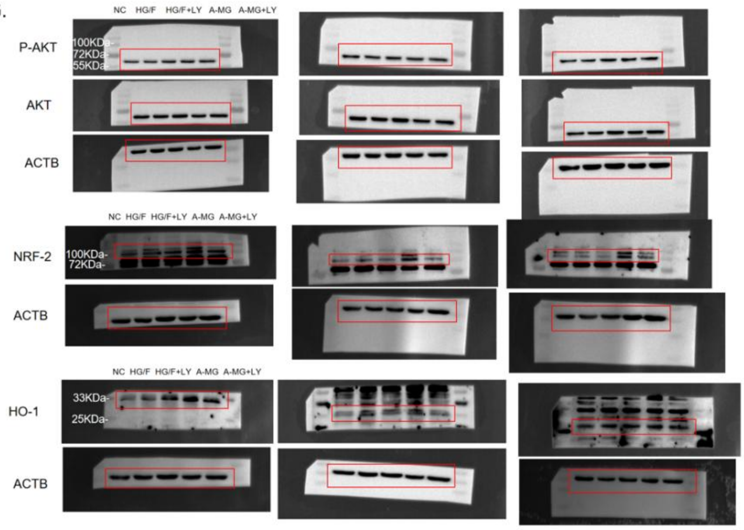

H.

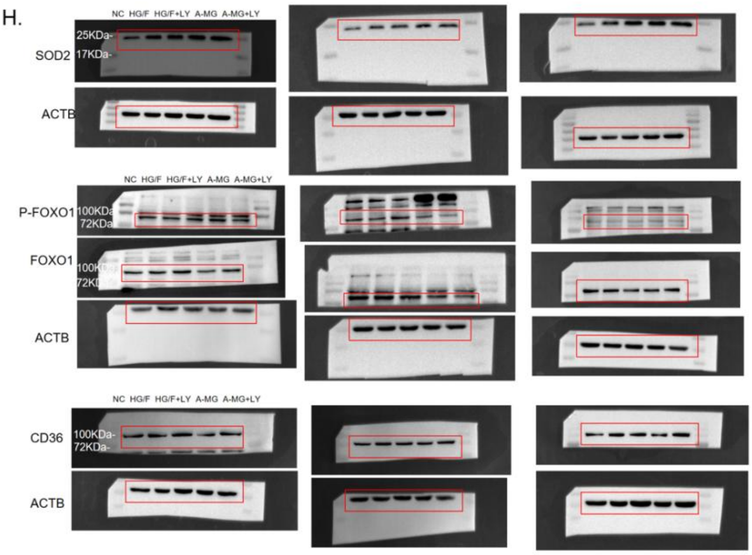

I.

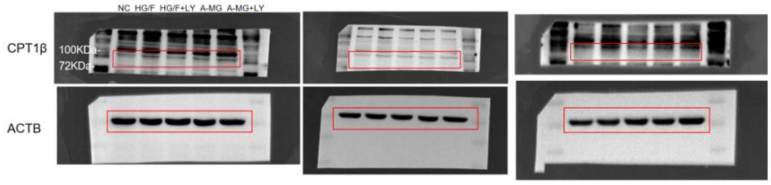

Figure S6 Western blot full size image of H9C2 cells. Figure **G-I** Shows the H9C2 cells were grouped into NC, HG/F, HG/F+LY294002, A-MG, A-MG+LY294002. Western blot analysis of the protein levels of P-AKT, AKT, NRF-2, HO-1, SOD2, FOXO1, CD36 and CPT1β, where HG/F+LY=HG/F+LY294002, A-MG+LY=A-MG+LY294002.

J.

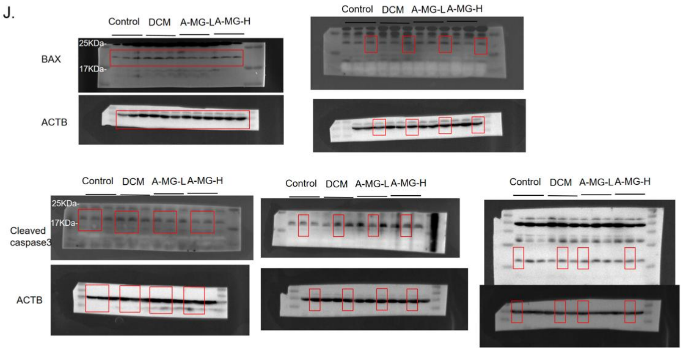

K.

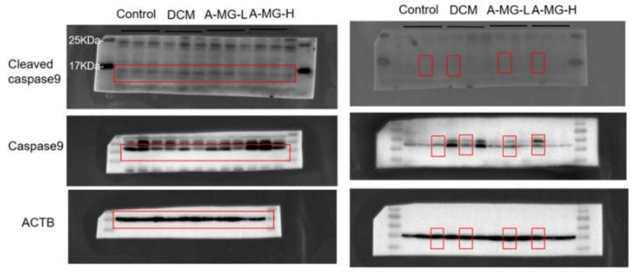

Figure S7 Full-size images of western blots of myocardial tissue of diabetic mice induced by HFD and STZ. Figure **J-K** Shows the mice were grouped into Control, DCM, A-MG-L, A-MG-H. The expression of apoptosis-related proteins BAX, Caspase3, Caspase9 were detected by Western blot.

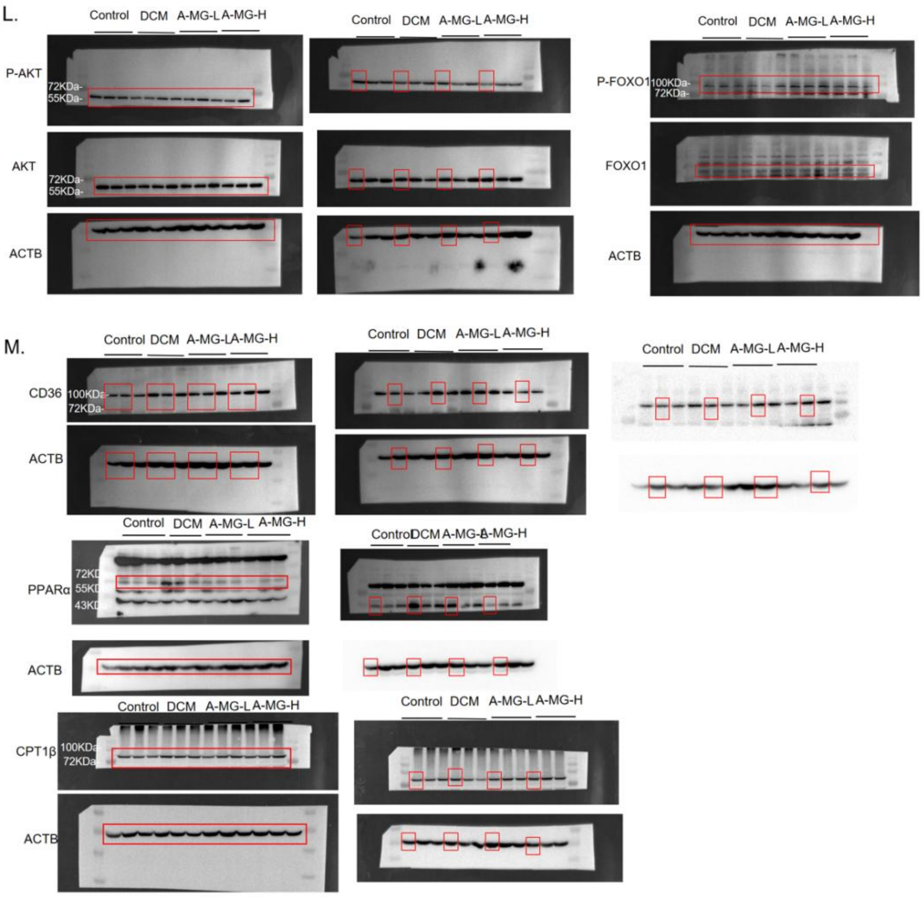

Figure S8 Full-size images of western blots of myocardial tissue of diabetic mice induced by HFD and STZ. Figure **L-M** Shows the mice were grouped into Control, DCM, A-MG-L, A-MG-H. Western blot analysis of P-AKT, AKT, P-FOXO1, FOXO1, CD36, PPARα, CPT1β protein expression.

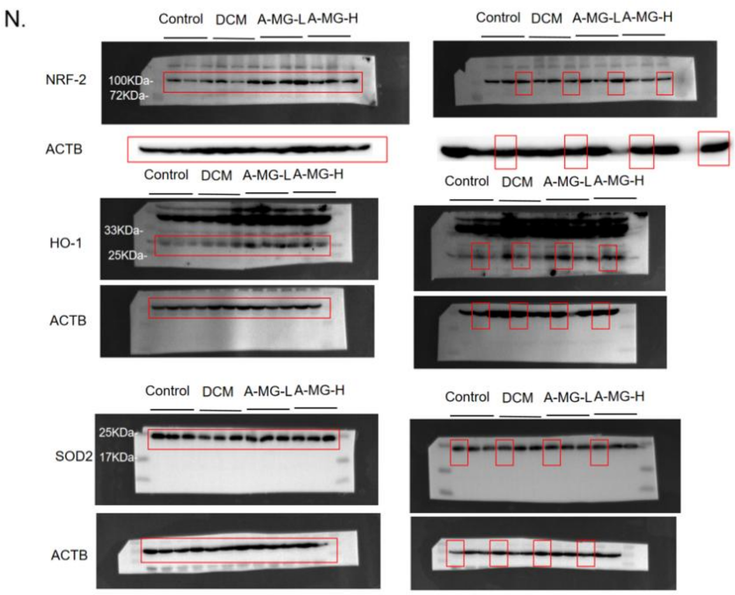

Figure S9 Full-size images of western blots of myocardial tissue of diabetic mice induced by HFD and STZ. Figure N Shows the mice were grouped into Control, DCM, A-MG-L, A-MG-H. Western blot analysis of NRF-2, HO-1, SOD2 protein expression.

In the process of electrical transfer of proteins, multiple proteins were transfected from different parts of the same SDS-PAGE gel using pre-stained protein marker as reference. In this process, we used membrane regeneration solution to incubate different antibodies in PVDF membrane again. We have provided three independent repeats and submitted the whole western blot picture as an attachment. In addition, we labeled the molecular weight of the protein in the figures.
